# A comparative analysis of the sensitivity and BOLD contamination of the VASO response at 3 Tesla: ME-DEPICTING vs. ME-EPI readouts ^†^

**DOI:** 10.1162/imag_a_00333

**Published:** 2024-10-28

**Authors:** Ratnamanjuri Devi, Jöran Lepsien, Toralf Mildner, Harald E. Möller

**Affiliations:** NMR Methods & Development Group, Max Planck Institute for Human Cognitive and Brain Sciences, Leipzig, Germany

**Keywords:** multi-echo EPI, center-out EPI, VASO, Δ *R*_2_^*^, blood, extravascular fraction, intravascular BOLD response

## Abstract

‘Non-BOLD fMRI’ data acquired at non-zero echo time (TE) suffer from contamination by the Blood Oxygenation Level Dependent (BOLD) signal due to the unavoidable signal decay caused by transverse relaxation. This contamination further reduces their already low inherent functional sensitivities and makes their correction essential. The Slice-Saturation Slab-Inversion Vascular Space Occupancy (SS-SI–VASO), for instance, cancels out BOLD contributions from VASO data, reflecting cerebral blood volume (CBV) changes, via a dynamic division approach. Alternatively, multi-echo (ME) data provide the possibility of extrapolating toTE=0. Acquisitions at very shortTEwould minimize the need for such corrections. The center-out EPI variant (‘DEPICTING’) is one such readout which allows for shortTE. The ME 2D DEPICTING was compared here against a traditional ME 2D EPI for its sensitivity to functional changes in the VASO signal. The two BOLD-correction schemes were also evaluated. Clear differences in functional sensitivity were observed for the uncorrected VASO data obtained from the first echo,${\text{TE}}_{1}$, of the two readouts. VASO data corrected by ME extrapolation were, however, found to be almost identical in their sensitivity for detecting CBV changes for both readouts. An excessively high increase in VASO signal sensitivity observed with the dynamic division correction for both readouts revealed a near-perfect linear dependence onTEof VASO signal changes. This could be attributed to the substantial intravascular BOLD contributions at 3 T. In the present data, extravascular$\Delta R_{2}^{*}$fraction was found to be around ~50–60%. ME extrapolation is, hence, recommended to avoid overestimation of functional CBV changes at commonly used TEs.

## 1 Introduction

VAscular Space Occupancy (VASO) functional magnetic resonance imaging (fMRI) provides indirect measures of changes in cerebral blood volume (CBV; in units of ml blood per ml of tissue) by nulling all blood signal at the time of acquisition (Lu et al., 2003), such that the VASO signal is related to the CBV according to (Huber, Ivanov, et al., 2014):

$$S_{\text{VASO}}\propto 1-\text{CBV.}$$ (1)

Since its inception in 2003, the technique has evolved into many variants, including Multiple Acquisitions with Global Inversion Cycling (MAGIC) VASO, which allowed for multi-slice and whole brain imaging (Lu, Van Zijl, et al., 2004;Scouten & Constable, 2007); inflow-based VASO (iVASO) for arterial and arteriolar quantitative CBV measurements (Hua et al., 2011); or Slab-selective Inversion (SI) VASO (Jin & Kim, 2008) and Slice-Saturation Slab-Inversion (SS-SI) VASO (Huber, Ivanov, et al., 2014) for improved sensitivity. Of these, SS-SI-VASO has caught the most traction in the past few years due to its applicability in high-resolution high-field (≥7 T) layer-fMRI studies (Huber et al., 2015). The higher sensitivity and temporal resolution of SS-SI-VASO can also be exploited at lower field strengths. Corresponding implementations, however, were delayed due to the lower demand for layer-fMRI studies at 3 T. There are at present only three published SS-SI-VASO studies at 3 T (Guidi et al., 2023;Huber, Kronbichler, et al., 2023;Knudsen et al., 2023).

Alternatives to blood oxygenation level-dependent (BOLD) fMRI techniques, such as VASO fMRI, also serve as proxies for neuronal activation and help supplement a more holistic understanding of BOLD fMRI. The irony, however, is in the fact that at non-zero echo time (TE), these non-BOLD methods themselves suffer from contamination by the BOLD response (Hetzer et al., 2011;Huber et al., 2019). The functional sensitivity and accuracy of these measurements could then benefit greatly from short-TE readouts. One such readout is the Double-shot Echo Planar Imaging with Center-out Trajectories and Intrinsic NaviGation or DEPICTING (Hetzer et al., 2011). The multi-echo (ME) version of DEPICTING with very short first TE (${\text{TE}}_{1}$) and inter-echo time was recently found to substantially reduce BOLD contamination in pseudo-Continuous Arterial Spin Labeling (pCASL) (Alsop et al., 2015;Dai et al., 2008;Lorenz et al., 2018) measurements of cerebral blood flow (CBF) changes, while providing reliable measures of the simultaneous BOLD response (Devi et al., 2022). SS-SI-VASO generally takes care of the inherent BOLD contamination at non-zero TE by employing a dynamic division strategy, wherein the blood-nulled image is divided by its consecutive non-nulled (BOLD-weighted) control image. A complete correction for BOLD contamination, however, relies on a number of factors, most importantly being the assumption of a similar BOLD contribution in both the*nulling*and*control*condition, that is, extravascular (EV) BOLD contributions being equivalent to those of the combined extravascular plus intravascular (IV) BOLD response in the non-nulled parenchyma (Huber, Ivanov, et al., 2014).

In the present study, the feasibility of ME-DEPICTING as a potential readout for SS-SI-VASO at 3 T was investigated. Owing to its shorter TEs, a higher functional sensitivity is expected, as compared to the ME variant of the commonly used Echo Planar Imaging (EPI). All assessments of ME-DEPICTING-based SS-SI-VASO were, hence, contrasted to those of ME-EPI-based SS-SI-VASO. Apart from the provision for simultaneous BOLD and CBV measurements, the ME readouts also allowed for a comparison of the BOLD-correction strategies. In particular, a correction provided by ME extrapolation toTE=0was tested against the correction provided by the original dynamic-division strategy.

## 2 Materials & Methods

### 2.1 Participants

Sixteen healthy volunteers (30 ± 5 years, 9 female) gave written informed consent before undergoing the experiments that had been approved by the Ethics Committee at the Medical Faculty of Leipzig University. All participants were right-handed and had normal or corrected-to-normal vision.

### 2.2 Functional paradigm

A full-field 8-Hz flickering black-and-white radial checkerboard was used for visual stimulation. Each functional cycle lasted 20 repetitions, starting with a rest block, which consisted of a blank gray screen of 12 repetitions (24 s), followed by the task block of 8 repetitions (16 s). The paradigm was programmed using Presentation (v17.2, Neurobehavioral Systems, Berkeley, CA, USA). A central, colored fixation point was present throughout the experiment. Subjects were instructed to focus on this dot and press a button whenever it changed color. Their attention was monitored by visually tracking their responses.

### 2.3 Magnetic resonance acquisitions

SS-SI-VASO was implemented on a 3-T MAGNETOM Skyra^fit^scanner (Siemens Healthineers, Erlangen, Germany) equipped with a 32-channel receive head coil. Data were obtained from 10 slices of 4 mm thickness (no slice gap, nominal in-plane resolution 3 mm × 3 mm, field of view 192 mm, matrix 64 × 64, bandwidth 2232 Hz/Px) located along the calcarine sulcus and acquired in descending slice order. Selective inversion for the VASO scans (nulling condition) was achieved over a 34-cm slab centered at the middle of the slice package through a hyperbolic secant inversion radiofrequency (RF) pulse of 8 ms duration, bandwidth-time product of 10, and RF field peak amplitude of$B_{1}$= 14 μT. The blood-nulling condition, corresponding to an inversion time, TI = 1153 ms, for an assumed longitudinal relaxation time of blood of$T_{1,b}=1664\text{ms}$(Lu, Clingman, et al., 2004), was fulfilled for the 6th slice. A schematic of the SS-SI-VASO implementation is given inFigure 1along with the sequence diagrams for ME-DEPICTING and ME-EPI. The specifications for the two two-dimensional (2D) ME readout modules were as follows:

**Fig. 1.**
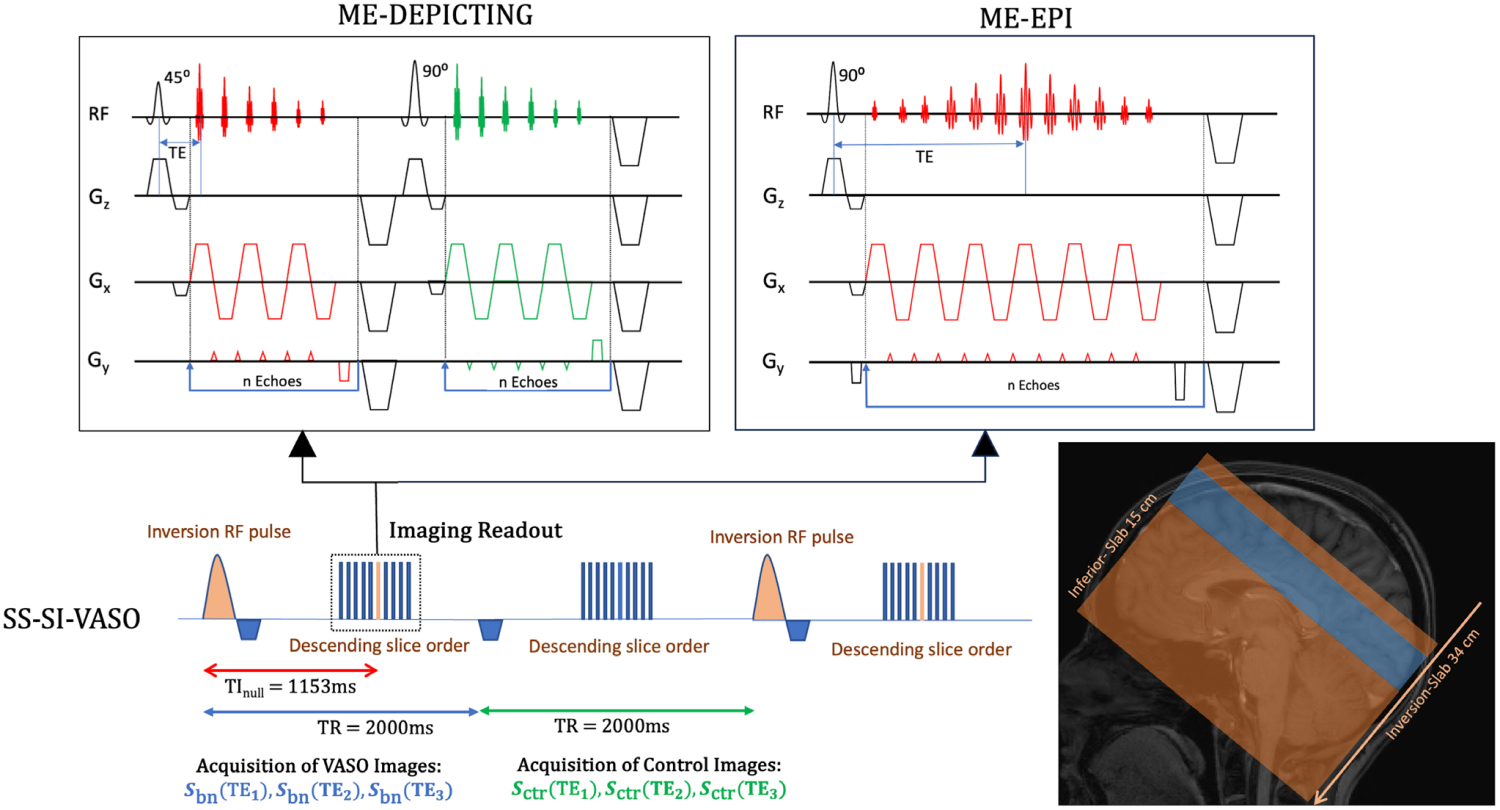
SS-SI-VASO implementation with ME-DEPICTING and ME-EPI as imaging readouts. For the slice package centered at the calcarine sulcus, the blood nulling condition at 3 T was fulfilled for the 6th slice (in orange) of the VASO images. The interleaved acquisition of VASO and Control images was accomplished with both ME-DEPICTING and ME-EPI readouts forn= 3 echoes. The sequence diagrams of the two readouts are provided. The double-shot nature of DEPICTING results in a shorter TE when compared to ME-EPI.

- ME-DEPICTING:${\text{TE}}_{1}$/${\text{TE}}_{2}$/${\text{TE}}_{3}$= 1.7 ms/10.7 ms/19.7 ms; repetition time,TR= 2 s; GRAPPA (GeneRalized Autocalibrating Partial Parallel Acquisition) factor 2.
- ME-EPI:${\text{TE}}_{1}$/${\text{TE}}_{2}$/${\text{TE}}_{3}$= 7.5 ms/20.7 ms/33.9 ms;TR = 2 s; GRAPPA factor 2; partial-Fourier factor 6/8.

Functional runs consisted of ten functional cycles (200 repetitions, i.e., 100 nulling/control condition pairs; effective TR,${\text{TR}}_{\text{eff}}$= 4 s) and were of approximately 7 min duration. ME-EPI and ME-DEPICTING acquisitions were recorded during the same session. Their order was shuffled across participants to avoid primacy bias.

In addition to these two functional runs, two auxiliary functional runs of similar duration were recorded in one of the subjects, with ME-EPI and ME-DEPICTING specifications different from those stated above. They were:

(i) ME-EPI with 5 echoes:${\text{TE}}_{1}$/${\text{TE}}_{2}$/${\text{TE}}_{3}$/${\text{TE}}_{4}$/${\text{TE}}_{5}$= 10 ms/25.3 ms/40.6 ms/55.9 ms/71.2 ms;TR= 2 s; GRAPPA factor 2; partial-Fourier factor 7/8.
(ii) High-resolution ME-DEPICTING: 15 slices, 1.7 mm isotropic nominal resolution;${\text{TE}}_{1}$/${\text{TE}}_{2}$/${\text{TE}}_{3}$= 2.8 ms/15.1 ms/27.4 ms;TR= 2 s; GRAPPA factor 2.

For co-registration purposes, a 2D spoiled gradient-recalled echo (GRE) scan (1.5 mm nominal in-plane resolution;TE= 3.1 ms;TR= 1300 ms; flip angle 90°) was obtained at the start of each session, with slice geometry identical to the functional scans.

### 2.4 Data preprocessing and analysis

Data were preprocessed and analyzed using Statistical Parametric Mapping (SPM12; Wellcome Trust Centre for Neuroimaging, UCL, London, UK) implemented in MATLAB 2022b (The MathWorks, Natick, MA, USA), FMRIB Software Library (FSL, (Jenkinson et al., 2012)), and additional scripts written in Interactive Data Language (IDL 8.1, Exelis Visual Information Solutions, Boulder, CO, USA).

#### 2.4.1 Preprocessing

All functional data were preprocessed in an identical manner. The data were split into VASO and BOLD datasets using ‘*fslsplit’*and ‘*fslmerge’*. The series acquired at${\text{TE}}_{1}$of each dataset was realigned, and the resulting realignment parameters applied to the corresponding remaining echoes. To allow for a meaningful comparison of functional sensitivity between the two readouts, only participants who exhibited minimal motion, defined as translation <1 mm and rotation <0.015 radians in both scans, were included in the analysis. The mean correlations between the realignment parameters of the VASO and BOLD scans in these subjects were also evaluated. The realigned time series were temporally high-pass filtered at a cut-off frequency equal to two effective functional cycles [1/(20$\times {\text{TR}}_{\text{eff}}$_)_] and 3D-Gaussian filtered at a full width at half maximum (FWHM) equal to the nominal voxel size (3 mm or 1.7 mm). Extrapolated ME signals at TE = 0,$S_{\text{bn}}\left(\text{TE}\to 0\right)$(please refer^1^) and$S_{\text{ctr}}\left(\text{TE}\to 0\right)$, as well as the effective transverse relaxation rates,$R_{2,\text{bn}}^{*}$and$R_{2,\text{ctr}}^{*}$, of the VASO and BOLD datasets, respectively, were then extracted from the preprocessed ME image volumes acquired at${\text{TE}}_{i}$with voxel intensity$S\left({\text{TE}}_{i}\right)$via linear regression (LINFIT) of the expression$\ln S\left({\text{TE}}_{i}\right)=-{\text{TE}}_{i}\times R_{2}^{*}+lnS\left(\text{TE}\to 0\right)$.

#### 2.4.2 Parameters

Percent VASO signal changes were then evaluated from: (i)*uncorrected*first echo of the blood-nulled dataset,$S_{\text{bn}}({\text{TE}}_{1})$, (ii) blood-nulled data of all echoes corrected by dynamic division, such that$S_{\text{dd}}\left({\text{TE}}_{i}\right)$$=S_{\text{bn}}\left({\text{TE}}_{i}\right)/S_{\text{ctr}}\left({\text{TE}}_{i}\right)$, and (iii) the ME-extrapolated intercept,$S_{\text{bn}}\left(\text{TE}\to 0\right)$. The simultaneously acquired BOLD signal was evaluated (i) in terms of$\Delta R_{2}^{*}$from the$R_{2,\text{bn}}^{*}$and$R_{2,\text{ctr}}^{*}$regression results, and (ii) in terms of percent BOLD signal changes from the weighted summation of all echoes,$S_{\text{ctr}}\left(\text{sum}\right)$(Poser et al., 2006;Posse et al., 1999). The weights were computed from$S_{\text{ctr}}\left({\text{TE}}_{i}\right)$and the underlying BOLD model according to the fitted$R_{2,\text{ctr}}^{*}$of each voxel (seeEq. 6ofPoser et al., 2006).

#### 2.4.3 Analysis

A general linear model (GLM) with the canonical hemodynamic response function (double gamma function) was implemented in IDL for statistical analyses and applied to all data.

#### 2.4.4 Quantification

Activation-related percent signal changes were quantified based on the resultingβcoefficients, such that$\delta s=\left[\left(\beta_{1}-\beta_{0}\right)/\beta_{0}\right]100\%$. Percent VASO signal changes were converted into relative changes in cerebral blood volume by assuming a resting value of${\text{CBV}}^{\text{rest}}$= 0.05 ml/ml and negligible contribution from cerebrospinal fluid (CSF) (Scouten & Constable, 2007). The imperfect blood nulling in the remaining slices was then compensated based onEq. 2btherein, rewritten here as:

$$\delta \text{cbv}=\frac{\Delta \text{CBV}}{{\text{CBV}}^{\text{rest}}}\times 100\text{\%}=\left(1-Q\frac{\rho_{\text{EV+IV}}}{\rho_{\text{IV}}{\text{CBV}}_{\text{rest}}}\right)\delta s_{\text{VASO}},$$ (2)

with$Q={\left(1-M_{z,\text{IV}}\left(\text{TI}\right)/M_{z,\text{EV}}\left(\text{TI}\right)\right)}^{-1}$for non-nulled slices;Q=1for the nulled slice; and water densities of blood,$\rho_{\text{IV}}=$0.86 ml water/ml blood, and parenchyma,$\rho_{\text{EV}+\text{IV}}=$0.89 ml water/ml parenchyma.$M_{z,\text{IV}}\left(\text{TI}\right)$and$M_{z,\text{EV}}\left(\text{TI}\right)$are the longitudinal magnetizations per unit of water in blood and parenchyma, respectively, at the time of acquisition; andΔCBVis the absoluteCBVchange in ml blood/ml of parenchyma. For calculation of the quantitative rate changes$\Delta R_{2}^{*}$in units of s^−1^,$R_{2}^{*,\text{rest}}$was taken directly from the estimated$\beta_{0}$parameter of the respective$R_{2}^{*}$time series.

#### 2.5 Sensitivity of VASO and BOLD signal changes

Significant regions of VASO and BOLD activation were identified based on a voxel-based significance threshold of$p<10^{-6}$, except for the high-resolution data, which owing to the lower signal to noise ratio (SNR) was thresholded at$p<10^{-4}$. Slices cropped during the realignment process due to inter-session motion were disregarded from all scans of the particular session.

The sensitivity of the VASO response was evaluated from$S_{\text{bn}}({\text{TE}}_{1})$,$S_{\text{dd}}\left({\text{TE}}_{i}\right)$and$S_{\text{bn}}\left(\text{TE}\to 0\right)$, while that of the BOLD response was evaluated from$R_{2,\text{ctr}}^{*}$and$S_{\text{ctr}}\left(\text{sum}\right)$. The comparison of$S_{\text{dd}}\left({\text{TE}}_{i}\right)$and$S_{\text{bn}}\left(\text{TE}\to 0\right)$VASO responses, inadvertently, allowed a comparison of the two BOLD correction strategies. The functional sensitivities were assessed in terms of (i) number of suprathreshold voxels, and (ii) the more reliable metric of temporal CNR (Geissler et al., 2007). The latter was evaluated from the common region of interest (ROI) between the ‘VASO activation’ obtained from the ME-extrapolated$S_{\text{bn}}\left(\text{TE}\to 0\right)$and the ‘BOLD activation’ taken from the$R_{2,\text{ctr}}^{*}$images of both readouts. Comparisons of relative VASO or BOLD signal changes across TEs were also based on this ROI.

#### 2.6 Extravascular and intravascular BOLD contributions

The parenchymal and extravascular BOLD contributions were estimated to evaluate the BOLD correction by dynamic division, which relies on the assumption$T_{2,\text{EV}+\text{IV}}^{*}$≈$T_{2,\text{EV}}^{*}$. The intravascular BOLD contribution was then estimated in terms of$\Delta R_{2}^{*}$, written as$\Delta R_{2,\text{IV}}^{*}$. These analyses were based on two ROIs in the nulled slice (i.e., the 6th/8th slice for standard-/high-resolution data):

(i) ROI-1 was based on common BOLD activation in the$R_{2,\text{ctr}}^{*}$data of the two readouts.$\Delta R_{2}^{*}$evaluated from$R_{2,\text{ctr}}^{*}$was expected to represent parenchymal$\Delta R_{2}^{*}$and is henceforth written as Δ$R_{2,\text{EV}+\text{IV}}^{*}$.
(ii) ROI-2 was based on common BOLD activation in the$R_{2,\text{bn}}^{*}$data of the two readouts. Δ$R_{2}^{*}$evaluated from$R_{2,\text{bn}}^{*}$was expected to represent extravascular$\Delta R_{2}^{*}$and is henceforth written as Δ$R_{2,\text{EV}}^{*}$.

The related effective transverse relaxation times during the resting condition,$T_{2,\text{EV+IV}}^{*,\text{rest}}$and$T_{2,\text{EV}}^{*,\text{rest}}$, were also extracted. Prior to the extraction of the blood-nulled slice from the visual cortex masks (-*fslslice*), the multi-slice mask was multiplied by a brain mask that had been extracted from the structural image (*-bet*with*-f 0.2*) (Smith, 2002) and linearly registered with the$S_{\text{bn}}\left(\text{TE}\to 0\right)$images of each readout (*-flirt*with*-applyxfm*,*-usesqform*& -*noresampblur*) (Jenkinson et al., 2002;Jenkinson & Smith, 2001). The relative extravascular rate change was evaluated from ROI-1 and ROI-2, such that

$$f_{\text{EV}}=\frac{\Delta R_{2,\text{EV}}^{*}}{\Delta R_{2,\text{EV+IV}}^{*}}\times 100,$$ (3)

The effective intravascular BOLD contribution of both readouts was estimated solely from ROI-2 according to:

$$\Delta R_{2,\text{IV}}^{*}=R_{2,\text{IV}}^{*,\text{act}}-R_{2,\text{IV}}^{*,\text{rest}}=-\frac{\ln E_{2,\text{IV}}^{\text{act}}}{\text{TE}}-R_{2,\text{IV}}^{*,\text{rest}},$$ (4)

An expression for$E_{2,\text{IV}}^{\text{act}}\equiv \exp\left(-\text{TE}\times R_{2,\text{IV}}^{*,\text{act}}\right)$is derived in theAppendix A1(Eq. A6). Experimental values of$T_{2,\text{EV}}^{*,\text{rest}}$and$T_{2,\text{EV}}^{*,\text{act}}$were taken from mean values in ROI-2. Similarly,${\text{CBV}}^{\text{act}}$was calculated from$\delta s_{\text{VASO},\text{bn}}\left(\text{TE}\to 0\right)$and the VASO signal from the dynamically divided middle echo$\delta s_{\text{VASO,dd}}\left({\text{TE}}_{\text{mid}}\right)$with${\text{TE}}_{\text{mid}}{\text{= TE}}_{2}$= 10.7 ms and 20.7 ms for ME-DEPICTING and ME-EPI, respectively, and${\text{TE}}_{\text{mid}}={\text{TE}}_{3}$= 40.6 ms for the ME-EPI with 5 echoes. The other relevant parameters had the following assumed values:$T_{1,\text{EV}}$= 1330 ms (Wansapura et al., 1999); relative proton densities,$\rho_{\text{IV}}$= 0.87 ml water/ml blood;$\rho_{\text{EV+IV}}$= 0.89 ml water/ml parenchyma (Donahue et al., 2009;Herscovitch & Raichle, 1985;Lu et al., 2002).$\Delta R_{2,\text{IV}}^{*}$was estimated for$T_{2,\text{IV}}^{*,\text{rest}}$varying from 15 ms to 40 ms in steps of 5 ms, that is from a more venous to a more arterial regime (Zhao et al., 2007). The influence of resolution and longer TE on$\Delta R_{2,\text{IV}}^{*}$and its dependence on$T_{2,\text{IV}}^{*,\text{rest}}$was also examined for a single subject with standard versus high-resolution ME-DEPICTING data and 3-echoes versus 5-echoes ME-EPI data.

## 3 Results

Four participants were excluded from the evaluation based on the motion criterion and another one due to minimal activation.Supplementary Figure S1shows examples of plots of the translational and rotational displacements of an excluded and an included subject. The mean correlation between the realignment parameters of VASO and control scans over the remaining 11 participants averaged at 0.86 ± 0.07 and 0.86 ± 0.09 for EPI and DEPICTING scans, respectively (Supplementary Table S1).

### 3.1 Sensitivity comparison of the readouts

**Fig. 2.**
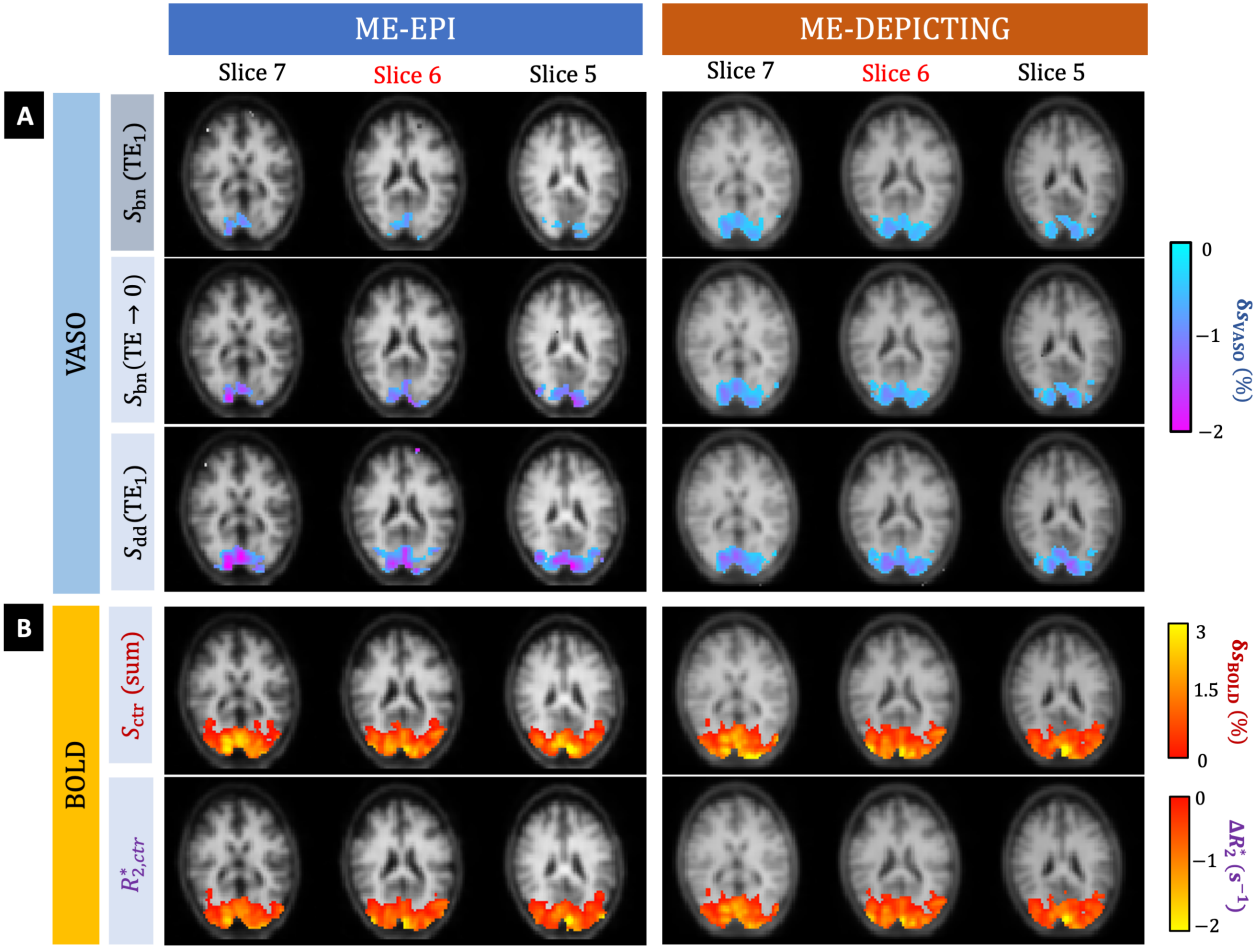
Simultaneously acquired VASO and BOLD responses in participant P1. (A) VASO activation maps obtained from the BOLD-uncorrected$S_{\text{bn}}\left({\text{TE}}_{1}\right)$, BOLD-corrected$S_{\text{bn}}\left(\text{TE}\to 0\right)$, and$S_{\text{dd}}\left({\text{TE}}_{1}\right)$. Note the larger activation areas obtained with ME-DEPICTING. (B) BOLD activation maps obtained from$S_{\text{ctr}}\left(\text{sum}\right)$and fitted$R_{2,\text{ctr}}^{*}$data. The$\delta {\text{s}}_{\text{VASO}}$(%),$\delta {\text{s}}_{\text{BOLD}}$(%), and$\Delta R_{2}^{*}$(s^–1^) in the activation maps, shown here for 3 slices, have been overlaid over the corresponding slices of the pre-processed$S_{\text{bn}}\left(\text{TE}\to 0\right)$of the two readouts.

**Fig. 3.**
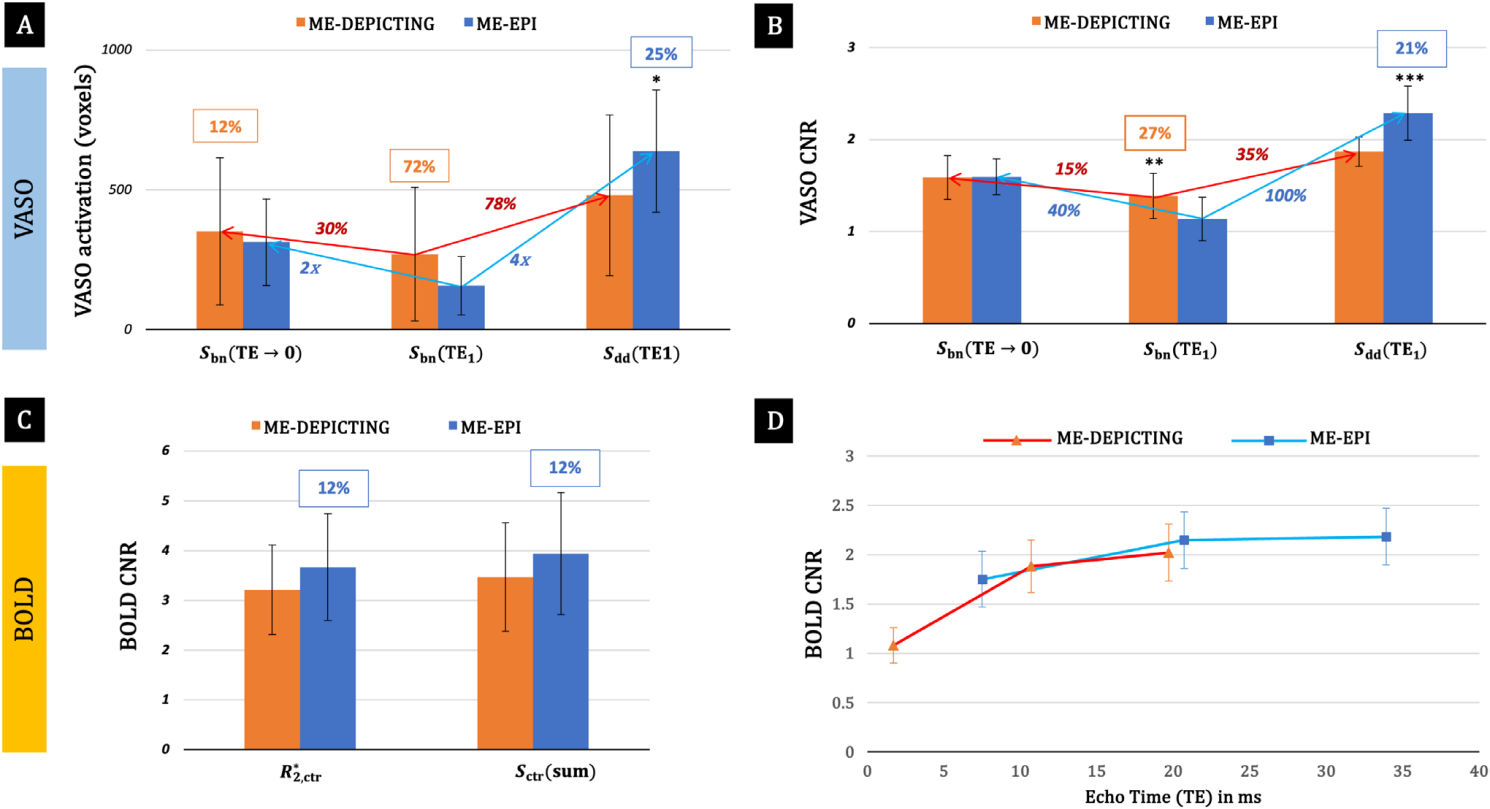
Functional sensitivity comparison of the two readouts (group-averaged data). Results obtained with the ME-DEPICTING readout are presented in orange and those with the ME-EPI readout in blue. Differences between the two readouts are given in % (orange and blue text boxes) and their significance is marked with asterisks (*, **, and *** forp<0.05,0.01, and0.001, respectively). Red and blue arrows indicate sensitivity gains for ME-DEPICTING and ME-EPI, respectively. Error bars denote standard deviations across participants. (A) Sensitivity for VASO signal detection: At minimumTE,DEPICTING yielded 72% more voxels than EPI. Correction for BOLD contamination with$S_{\text{bn}}\left(\text{TE}\to 0\right)$led to 30% and approx. 100% increases in the activated area for ME-DEPICTING and ME-EPI, respectively. Dynamic division yielded unexpectedly greater boosts of 78% and 300%, respectively. (B) VASO CNR in commonly activated regions of both readouts:$S_{\text{bn}}\left({\text{TE}}_{1}\right)$revealed a 27% higher CNR for DEPICTING compared to EPI (1.4 ± 0.3 vs. 1.1 ± 0.2). The CNR values obtained from the dynamically divided data were once again higher than those of the extrapolated data, yielding gains of 35% and 100% for ME-DEPICTING and ME-EPI, respectively. (C) BOLD CNRs based on$S_{\text{ctr}}\left(\text{sum}\right)$and$R_{2,\text{ctr}}^{*}$: The CNR of ME-EPI was only a 12% higher than that of ME-DEPICTING. (D)$S_{\text{ctr}}({\text{TE}}_{i}$) showing the expected increase of the BOLD sensitivity with TE for both readouts.

Significant VASO and BOLD activations were identified in all remaining participants. Examples are presented inFigure 2. The higher sensitivity of$S_{\text{bn}}\left({\text{TE}}_{1}\right)$of DEPICTING at the subject level (Fig. 2A) was confirmed for the group-averaged data (Fig. 3A). The number of significant voxels showing VASO, BOLD and their common activation (VASO∩BOLD, from$S_{\text{bn}}\left(\text{TE}\to 0\right)$and$R_{2,\text{ctr}}^{*},$respectively) for each participant can be assessed fromSupplementary Table S2. The temporal VASO CNR values are shown inFigure 3BandSupplementary Table S3. Interestingly, after extrapolation to zero TE, the activated areas of both readouts became rather similar and the CNR was almost equal.

It is to be noted that even though the ROI selection relied on BOLD activation from$R_{2,\text{ctr}}^{*}$(Supplementary Table S2), the sizes of the ROI were similar even when the significant BOLD area was based on$S_{\text{ctr}}\left(\text{sum}\right)$(not shown). The sensitivity difference for the detection of BOLD signal changes was found to be negligible (~7%) between the$S_{\text{ctr}}\left(\text{sum}\right)$and$R_{2,\text{ctr}}^{\text{*}}$for both readouts (Fig. 3CandSupplementary Table S4). The CNR of BOLD data obtained from each$S_{\text{ctr}}\left({\text{TE}}_{i}\right)$for the two readouts from the ROI is plotted inFigure 3D.

### 3.2 TE dependence of the BOLD correction by dynamic division

Investigation into the startling gain in VASO sensitivity of the dynamic division-corrected data revealed further increases in both the activation area (Supplementary Table S2) and VASO CNR (Supplementary Table S3) for$S_{\text{dd}}({\text{TE}}_{2}$) and$S_{\text{dd}}({\text{TE}}_{3}$) for both readouts. Significantly activated areas in two arbitrary participants are presented inFigure 4along with corresponding maps obtained from$S_{\text{bn}}\left(\text{TE}\to 0\right)$and$S_{\text{bn}}\left({\text{TE}}_{1}\right)$. However, in case of the dynamic-division method, along with the increase in activation area with TE, the intensity of the VASO signal change,$\delta s_{\text{VASO,dd}}$was also found to increase with TE. The increase of$\delta s_{\text{VASO,dd}}$withTEis also evident in their cycle-averaged time courses (green line with filled squares inFig. 5).

**Fig. 4.**
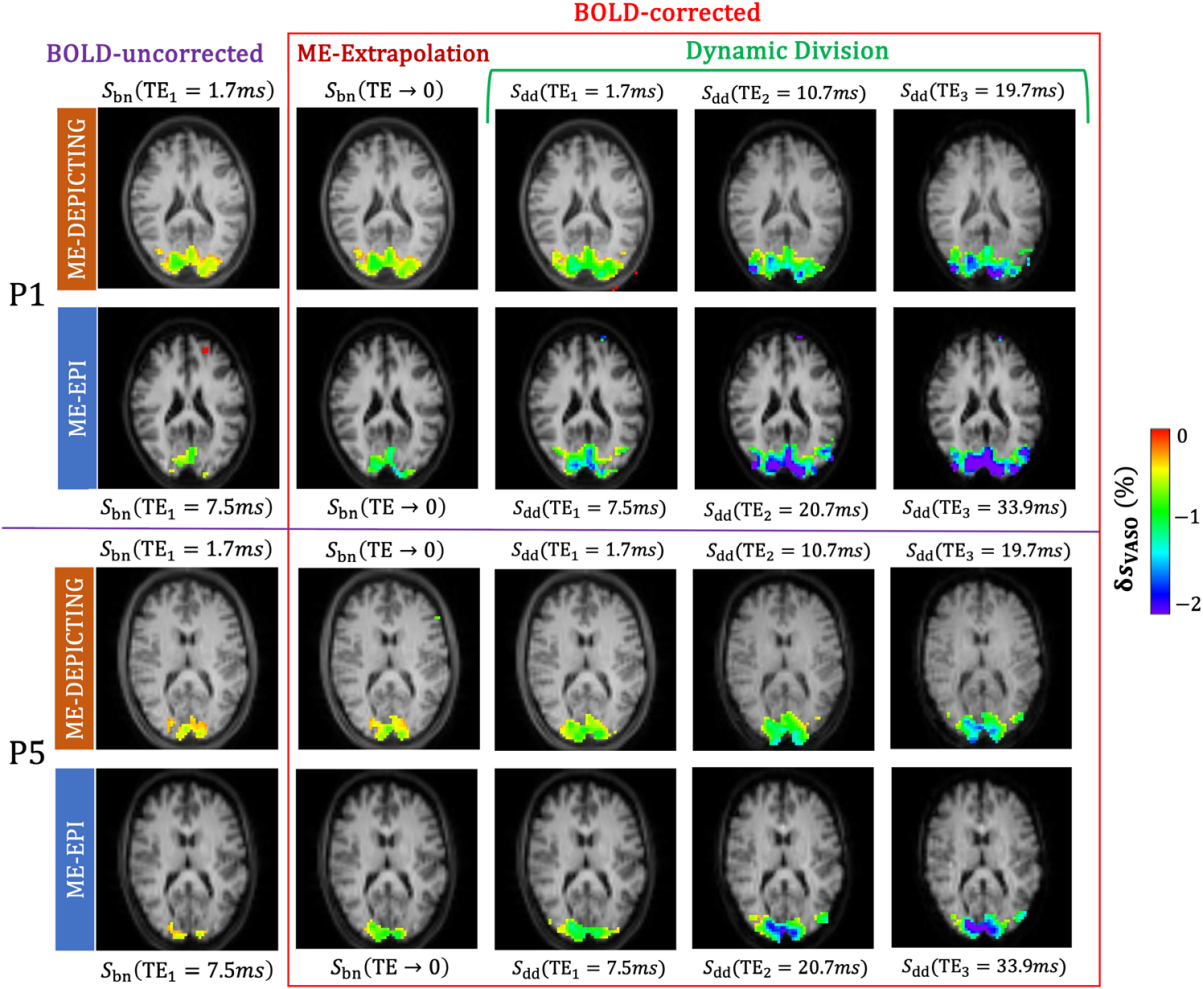
VASO activation maps obtained from the nulled slice in two participants (P1 and P5). The maps based on the$S_{\text{bn}}\left({\text{TE}}_{\text{1}}\right)$,$S_{\text{bn}}\left(\text{TE}\to 0\right)$and$S_{\text{dd}}({\text{TE}}_{i}$) data show BOLD-uncorrected$\delta s_{\text{VASO},\text{bn}}\left({\text{TE}}_{1}\right)$as well as BOLD-corrected results obtained by extrapolation to zeroTE,$\delta s_{\text{VASO,bn}}\left(\text{TE}\to 0\right)$, and by dynamic division,$\delta s_{\text{VASO,dd}}\left({\text{TE}}_{i}\right)$for all echo times. The activation maps have been overlaid over the 6th slice of the respective pre-processed VASO images at the specified TE. For both BOLD-correction strategies, the increase of the size and strength of activation compared to uncorrected data is evident. An increase in the intensity of the VASO signal change,$\delta s_{\text{VASO,dd}}$with TE is indicated by the growing blue/violet area for the$S_{\text{dd}}({\text{TE}}_{2}$) and$S_{\text{dd}}({\text{TE}}_{3}$) data of both readouts.

**Fig. 5.**
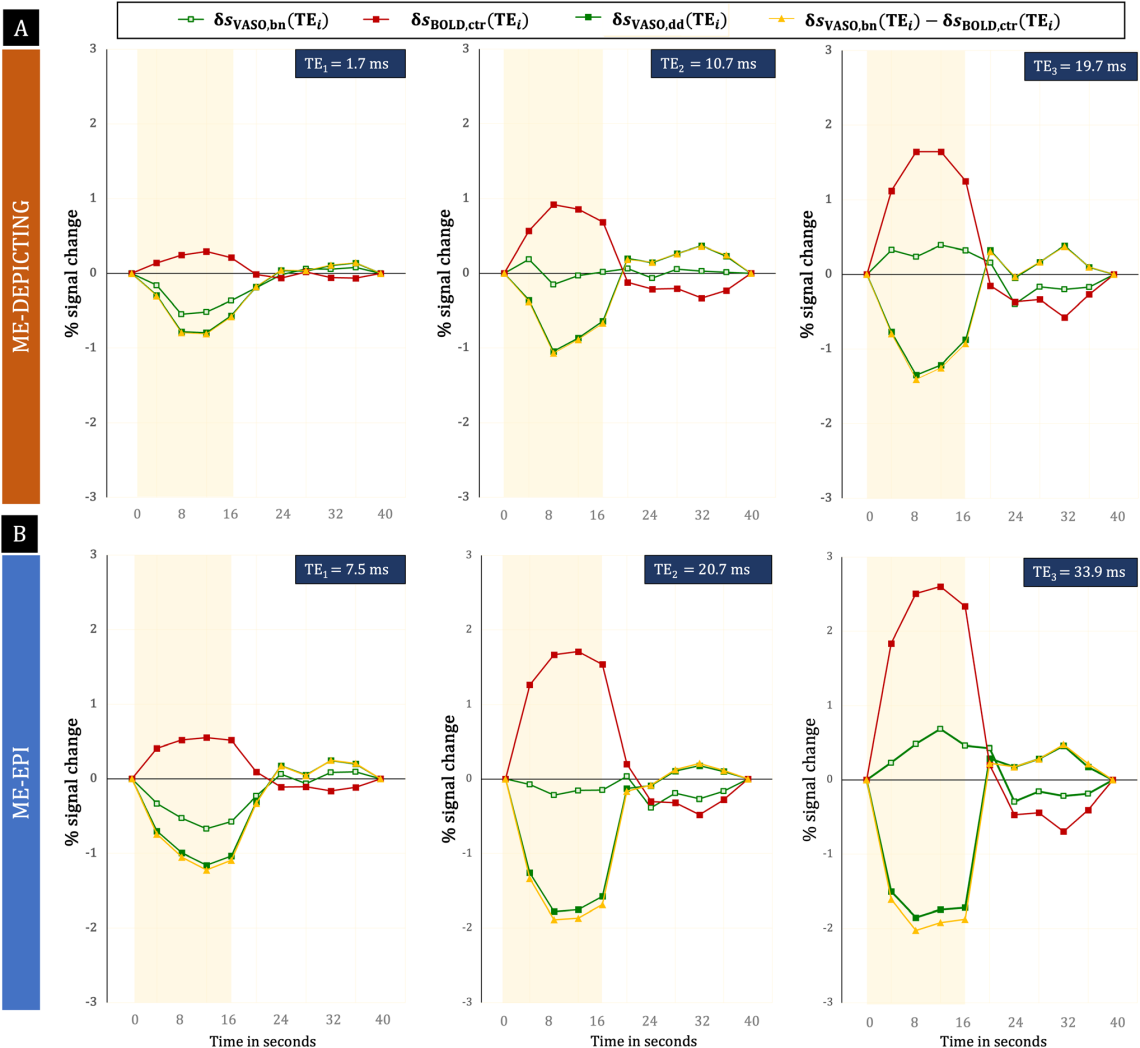
Cycle-averaged time courses of percent VASO signal changes. (A) ME-DEPICTING results, (B) ME-EPI results; both obtained in participant P1 (nulled slice; see top panel inFig. 4). Red squares: BOLD response from the control data,$\delta s_{\text{BOLD,ctr}}\left({\text{TE}}_{i}\right)$; open green squares: uncorrected VASO response,$\delta s_{\text{VASO,bn}}\left({\text{TE}}_{i}\right)$; filled green squares: VASO response corrected by dynamic division,$\delta s_{\text{VASO,dd}}\left({\text{TE}}_{i}\right)$. Yellow triangles represent the difference [$\delta s_{\text{VASO,bn}}({\text{TE}}_{i})-\delta s_{\text{BOLD,ctr}}({\text{TE}}_{i})]$. The yellow-shaded region denotes the stimulus duration.

The time courses inFigure 5were extracted separately for each readout from a common ROI of VASO activation in the nulled slices of ME-DEPICTING and ME-EPI data. As expected,$\delta s_{\text{VASO,bn}}\left({\text{TE}}_{i}\right)$switches from a negative to a positive response with increasingTEfor both readouts, while$\delta s_{\text{BOLD,ctr}}\left({\text{TE}}_{i}\right)$grows withTE, as evidenced by the amplitudes of the primary signals and post-stimulus undershoots. Interestingly*,*$\delta s_{\text{VASO,dd}}\left({\text{TE}}_{i}\right)$can be seen mirroring the corresponding BOLD signals. This applies to the evolution of both the signal intensities and the shapes of the time courses as a function ofTEfor both readouts. Another finding is that$\delta s_{\text{VASO,dd}}\left({\text{TE}}_{i}\right)$agreed rather well with the difference between the uncorrected VASO response during the nulling condition and the BOLD response during the control condition,$\delta s_{\text{VASO},\text{bn}}\left({\text{TE}}_{i}\right)-\delta s_{\text{BOLD},\text{ctr}}\left({\text{TE}}_{i}\right)$.

**Fig. 6.**
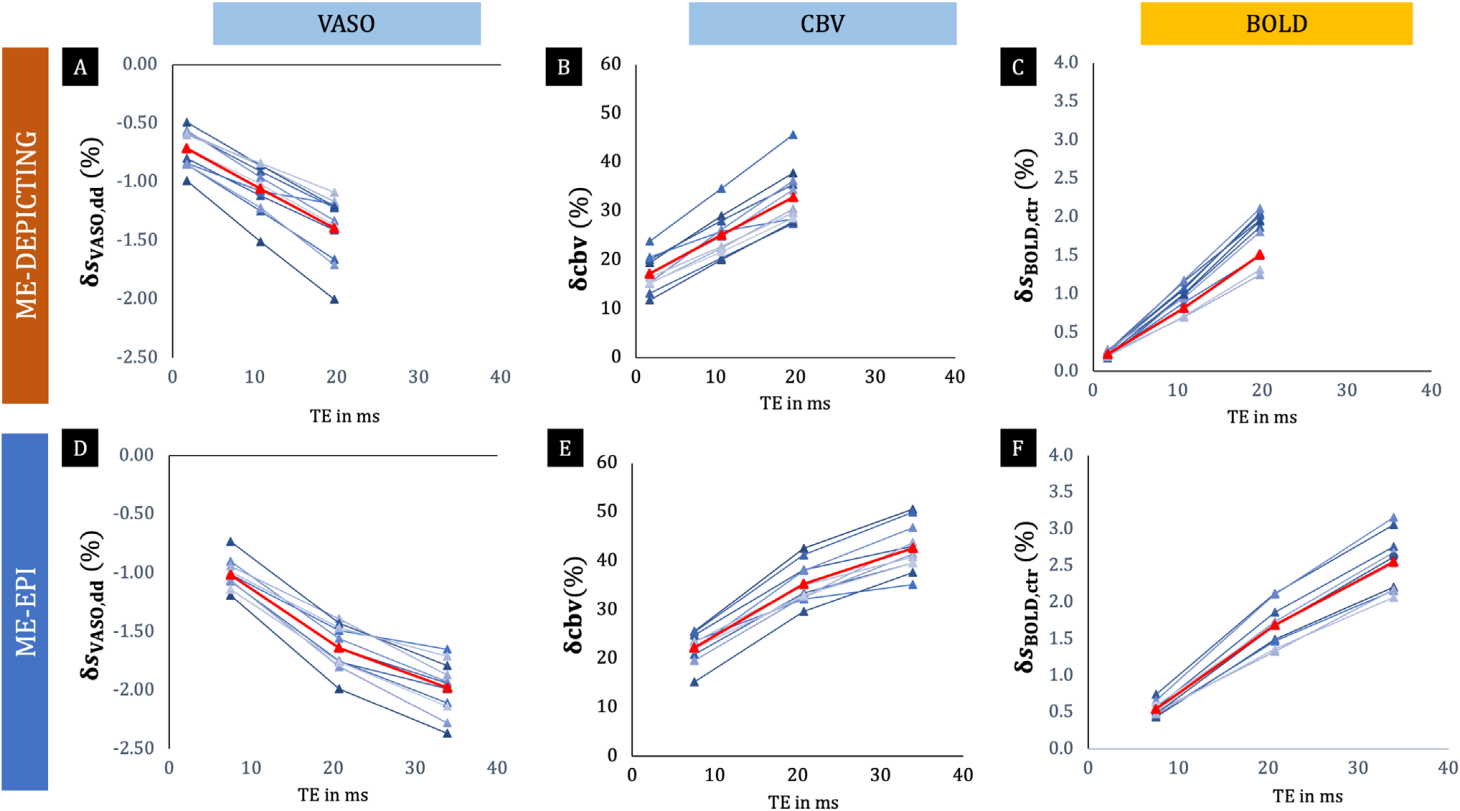
Echo-time dependence of dynamic-division corrected VASO signals. Mean$\delta s_{\text{VASO,dd}}$(A, D), calculated over the common ROI of VASO activation in the nulled slices of ME-DEPICTING (A) and ME-EPI (D), and the correspondingδcbv obtained withEq. 2(B, E) are plotted for all individual subjects against the corresponding${\text{TE}}_{i}$.$\delta s_{\text{BOLD,ctr}}$(C, F) serves as references for the TE dependence. The subject-averaged values are shown in red (also seeSupplementary Tables S5 and S6).

Figures 6and7further demonstrate the TE dependence of the dynamic division-corrected VASO data for both readouts. With a near-perfect linear increase ($r^{2}\geq 0.97$), the relation of$\left|\delta s_{\text{VASO,dd}}\right|$bears a striking resemblance to the TE dependence of$\delta s_{\text{BOLD,ctr}}$. The slopes of the linear fits of$\left|\delta s_{\text{VASO,dd}}\right|$and$\delta s_{\text{BOLD,ctr}}$averaged across subjects were found to be comparable between the two readouts.

**Fig. 7.**
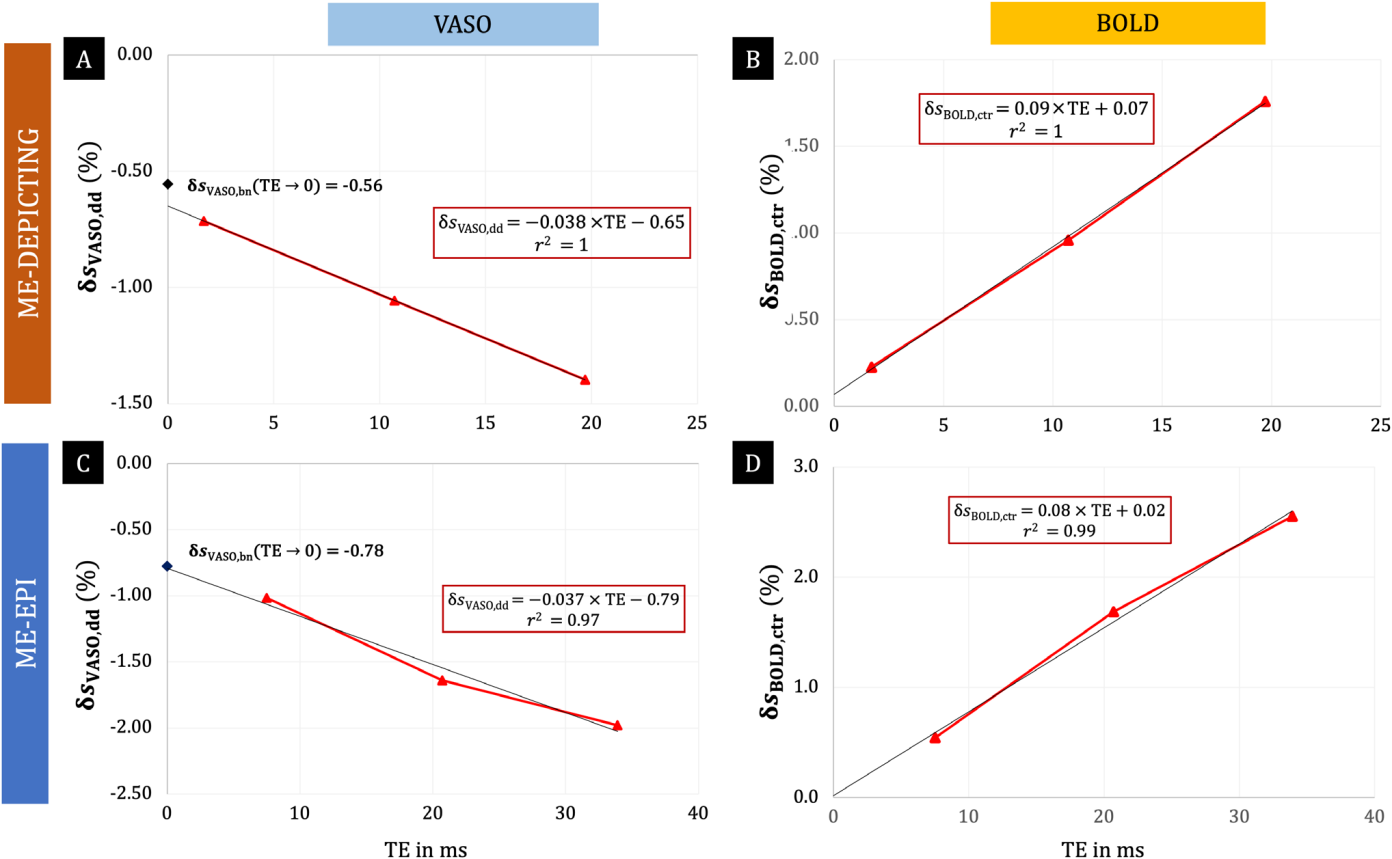
Linear relationships of subject-averaged VASO and BOLD responses withTE. Subject-averaged$\delta s_{\text{VASO,dd}}$(A, C) and$\delta s_{\text{BOLD, ctr}}$(B, D) were obtained from$S_{\text{dd}}\left({\text{TE}}_{i}\right)$and$S_{\text{ctr}}\left({\text{TE}}_{i}\right)$recorded with ME-DEPICTING (A, B) and ME-EPI (C, D). The linear relations and their corresponding goodness of fit,$r^{2}$, are given within the rectangular boxes. For comparison purposes, the subject-averaged δ$s_{\text{VASO,bn}}\left(\text{T}E\to 0\right)$values are indicated in (A) and (C) by black diamonds on they-axes. Residual BOLD signals extrapolated to zeroTEwere 0.02±0.14% and 0.07±0.03% for EPI and DEPICTING, respectively. These residual BOLD-like time-dependent signals and the corresponding$\delta s_{\text{VASO,bn}}\left(\text{TE}\to 0\right)$signals (for reference) for each individual participant are shown inSupplementary Figure S2.

**Fig. 8.**
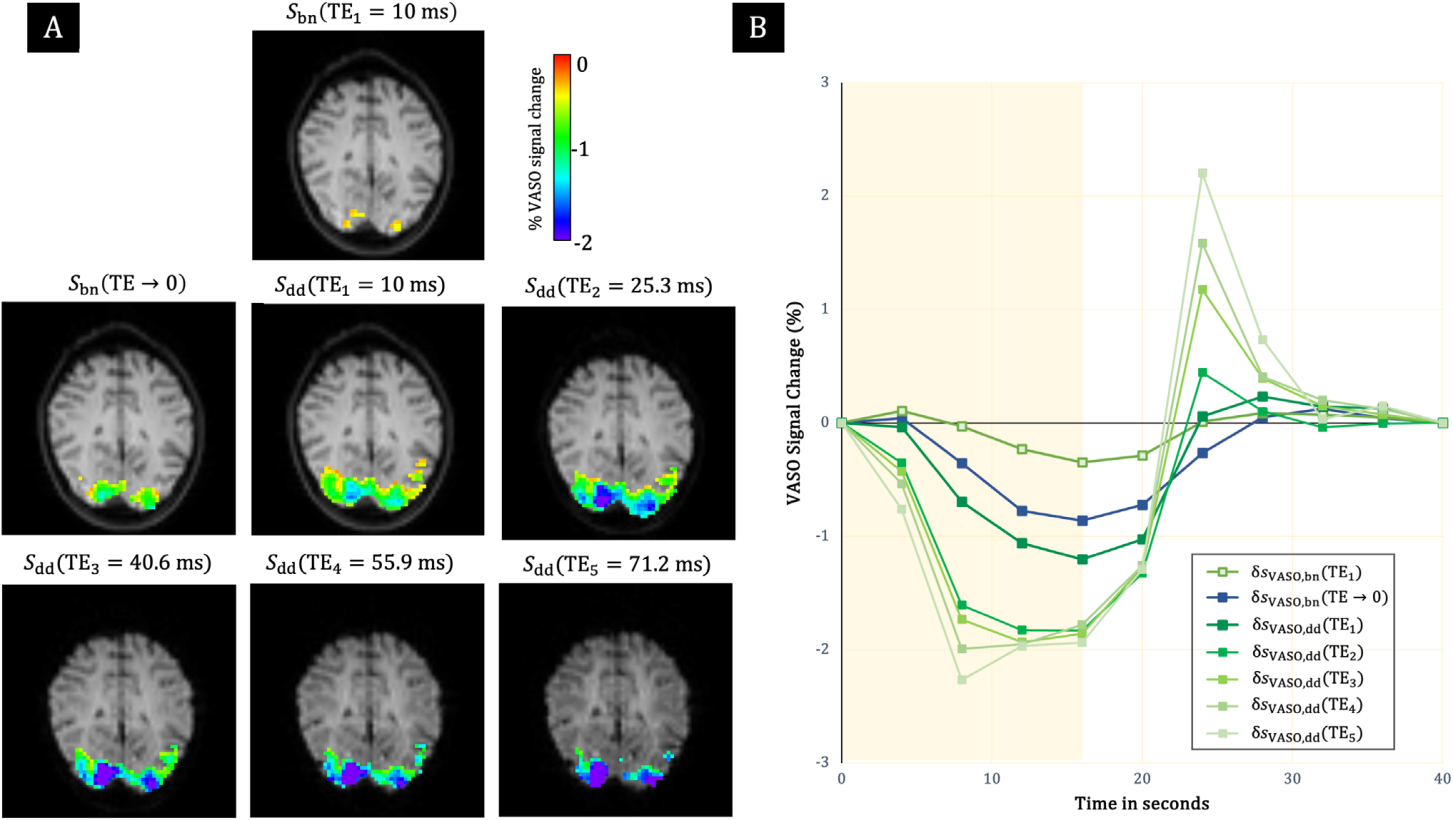
SS-SI-VASO acquisition with ME-EPI with 5 echoes. (A) Significant activation observed in the 6th slice (thresholded atp< 10^–6^) of the uncorrected VASO response from$S_{\text{bn}}\left({\text{TE}}_{\text{1}}\right)$, the BOLD-corrected VASO responses from the extrapolated$S_{\text{bn}}\left(\text{TE}\to 0\right)$, and the dynamically divided echoes$S_{\text{dd}}\left({\text{TE}}_{i}\right)$. The maps have been overlaid over the respective pre-processed VASO images at the specified TE. (B) Corresponding uncorrected ($\delta s_{\text{VASO,bn}}({\text{TE}}_{\text{1}}))$and corrected cycle-averaged time courses of$\delta s_{\text{VASO,bn}}\left(\text{TE}\to 0\right)$and$\delta s_{\text{VASO,dd}}\left({\text{TE}}_{i}\right)$for 1≤i≤5. The shaded yellow region denotes stimulus duration.

**Fig. 9.**
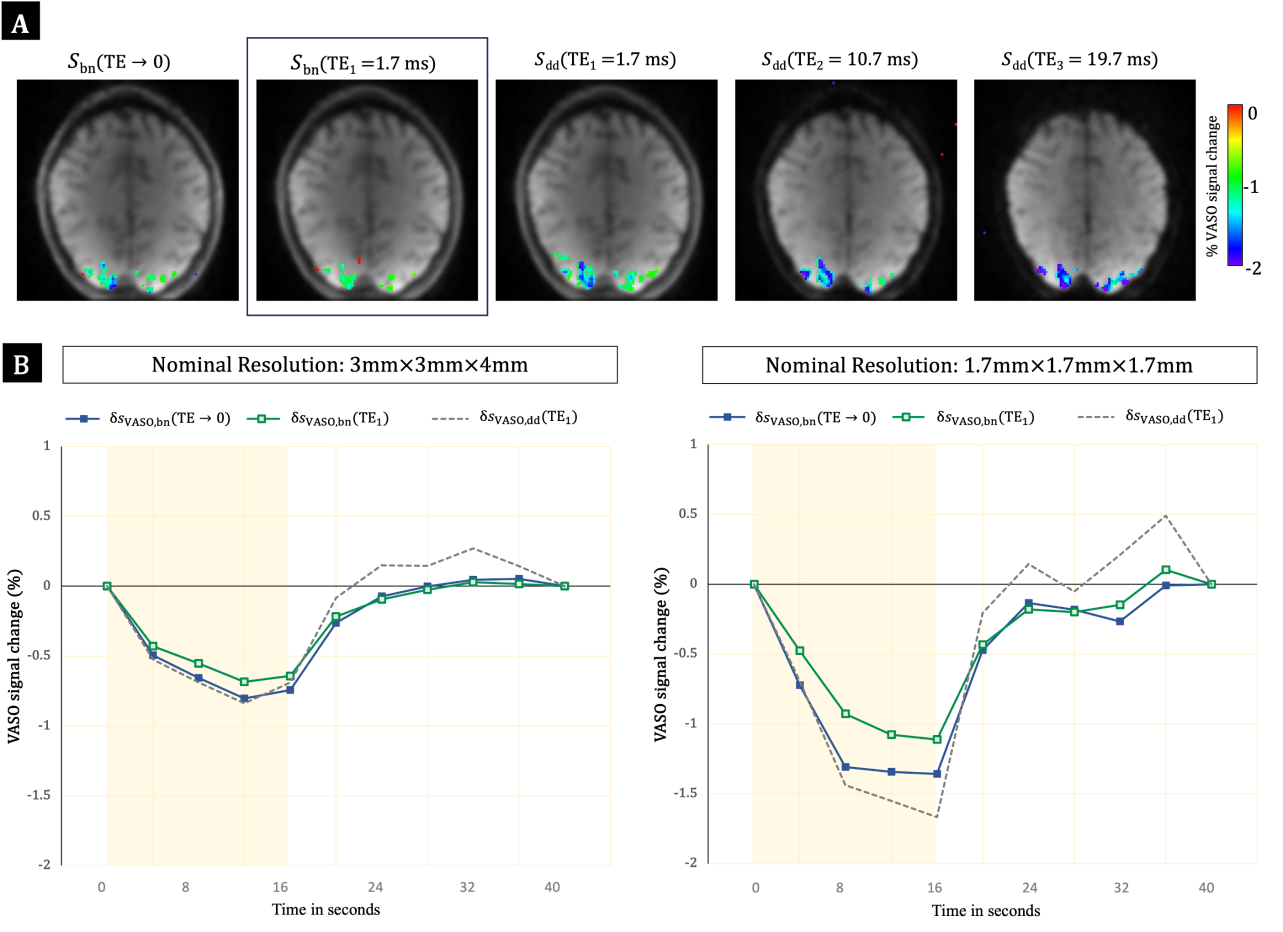
ME-DEPICTING SS-SI-VASO acquisition at higher resolution. (A) Significant VASO activation (p< 10^–4^) as seen on the nulled slice (8th slice) of the uncorrected$S_{\text{bn}}\left({\text{TE}}_{1}\right)$(2nd column, within the black square box), the ME-extrapolated$S_{\text{bn}}\left(\text{TE}\to 0\right)$(1st column), and the dynamically divided$S_{\text{dd}}\left({\text{TE}}_{i}\right)$(columns 3 to 5), overlaid over the corresponding unprocessed images. (B) Comparison of cycle-averaged time courses of VASO signal changes at different spatial resolutions:$\delta s_{\text{VASO,bn}}{\text{(TE}}_{\text{1}})$(in green),$\delta s_{\text{VASO,bn}}\left(\text{TE}\to 0\right)$(in blue), and$\delta s_{\text{VASO,dd}}{\text{(TE}}_{1})$(gray broken line). The signals were extracted from the region defined by activation in the$S_{\text{bn}}\left(\text{TE}\to 0\right)$data. Shaded yellow region denotes stimulus duration.

Furthermore, theTEdependence of$\delta s_{\text{VASO,dd}}$was also identified for the auxiliary single-subject data with 5 echoes (Fig. 8). A reduction in the extent of activation can be seen with later echoes (Fig. 8A). The amplitude of the primary signal of VASO responses grows rapidly at smaller TEs before appearing to stabilize atTE≥ 25.3 ms. A shift in the shape of the time courses can be seen for$\delta s_{\text{VASO},\text{dd}}\left({\text{TE}}_{i}\right)$; specifically, in a faster return to baseline compared to$\delta s_{\text{VASO},\text{bn}}\left({\text{TE}}_{1}\right)$and$\delta s_{\text{VASO},\text{bn}}\left(\text{TE}\to 0\right)$. An increase in the amplitude of the post-stimulus overshoot is also evident (Fig. 8B).Figure 9demonstrates a similar behavior of the dynamic division-corrected data (increase of$\delta s_{\text{VASO,dd}}$withTE) for higher-resolution data. The shape of the signals from$\delta s_{\text{VASO,dd}}{\text{(TE}}_{1})$deviates again from that of uncorrected$\delta s_{\text{VASO},\text{bn}}{\text{(TE}}_{1})$and ME-extrapolated$\delta s_{\text{VASO},\text{bn}}\left(\text{TE}\to 0\right)$. The larger amplitude of VASO signal changes at higher resolution is also evident.

### 3.3 Extra- and intravascular BOLD contributions

#### 3.3.1 Extravascular $\Delta {\text{R}}_{2}^{\text{*}}$ fraction

Averaged functional$\Delta R_{2,\text{EV}}^{\text{*}}$and$\Delta R_{2,\text{EV+IV}}^{\text{*}}$values resulted in estimates of the extravascular$\Delta R_{2}^{\text{*}}$fraction, over the parenchymal ROI-1 (146 ± 59 voxels), of$f_{\text{EV}}$= 55±15% and 50 ± 12% for ME-EPI and ME-DEPICTING, respectively. A substantial increase in this fraction was obtained in the smaller extravascular ROI-2 (45 ± 31 voxels):$f_{\text{EV}}$= 62 ± 14% and 60 ± 11%, respectively. The sizes of ROI-1 and ROI-2 for each participant and the corresponding$\Delta R_{2,\text{EV}+\text{IV}}^{\text{*}}$,$\Delta R_{2,\text{EV}}^{\text{*}}$and$f_{\text{EV}}$are provided inTable 1for both readouts along with the$T_{2,\text{EV+IV}}^{*,\text{rest}}$and$T_{2,\text{EV}}^{*,\text{rest}}$values.$T_{2,\text{EV}}^{*,\text{rest}}$was found to be slightly shorter than their$T_{2,\text{EV+IV}}^{*,\text{rest}}$counterparts with both EPI and DEPICTING in both ROIs. An example of the voxel-wise distribution of$T_{2,\text{EV+IV}}^{*,\text{rest}}$,$T_{2,\text{EV}}^{*,\text{rest}}$,$\Delta R_{2,\text{EV+IV}}^{*}$,$\Delta R_{2,\text{EV}}^{*}$and the extravascular$\Delta R_{2}^{*}$fraction over ROI-1 (participant P16: average$f_{\text{EV}}$of 51% and 46% for EPI and DEPICTING, respectively) is shown inFigure 10.

**Table 1.** Values of$T_{2}^{*,\text{rest}}$and$\Delta R_{2}^{*}$in the EV+IV and EV compartments (obtained from time series of$R_{2,\text{ctr}}^{*}$and$R_{2,\text{bn}}^{*}$, respectively) and the corresponding extravascular$\Delta R_{2}^{*}$fraction. Results obtained with both readouts are shown separately for ROI-1 and ROI-2 located in the blood-nulled slice.

Participant	ROI size	Parenchyma				GM					
		$T_{2,\text{EV+IV}}^{*,\text{rest}}$ (ms)		$\Delta R_{2,\text{EV+IV}}^{*}$ (s ^–1^ )		$T_{2,\text{EV}}^{*,\text{rest}}$ (ms)		$\Delta R_{2,\text{EV}}^{*}$ (s ^–1^ )		Extravascular $\Delta R_{2}^{*}$ fraction $f_{\text{EV}}$ (%)	
		EPI	DEPICTING	EPI	DEPICTING	EPI	DEPICTING	EPI	DEPICTING	EPI	DEPICTING
*ROI-1: Slice 6 of functional parenchymal visual cortex mask*											
P1	270	41.9	36.3	–0.69	–0.83	40.9	33.9	–0.35	–0.45	50.7	54.3
P3	195	41.2	40.1	–0.69	–0.96	39.7	38.6	–0.45	–0.53	71.2	56.1
P4	98	40.5	38.1	–0.81	–0.91	39.7	36.3	–0.50	–0.56	61.0	61.3
P5	141	48.5	42.4	–0.53	–0.74	46.4	39.8	–0.18	–0.34	33.0	44.6
P9	157	47.5	42.6	–0.46	–0.67	45.8	39.7	–0.36	–0.46	78.3	68.7
P10	130	42.4	36.2	–0.40	–0.78	42.3	34.9	–0.21	–0.44	53.6	53.4
P11	55	47.5	40.1	–0.52	–0.77	49.0	36.7	–0.41	–0.45	78.4	57.1
P12	163	52.1	46.5	–0.68	–0.58	49.2	42.0	–0.28	–0.15	42.6	25.9
P13	158	48.9	46.6	–0.50	–0.52	47.5	43.2	–0.25	–0.24	51.0	42.0
P14	75	47.2	45.0	–0.47	–0.57	45.5	42.7	–0.17	–0.26	38.5	44.6
P16	164	49.7	43.6	–0.45	–0.51	48.5	40.9	–0.23	–0.25	51.2	45.6
Mean ± SD	146 ± 59	46.1 ± 3.9	41.6 ± 3.7	–0.56 ± 0.13	–0.71 ± 0.16	45 ± 3.7	39 ± 3.2	–0.31 ± 0.11	–0.38 ± 0.13	55 ± 15	50 ± 12
*ROI-2: Slice 6 of functional extravascular visual cortex mask*											
P1	71	41.5	33.4	–0.86	–1.04	41.4	31.4	–0.49	–0.62	59.4	60.7
P3	97	41.5	42.3	–0.87	–1.16	39.3	39.9	–0.58	–0.73	70.1	63.5
P4	74	41.7	40.6	–0.88	–0.98	41.2	39.2	–0.58	–0.67	68.7	60.4
P5	17	50.1	48.1	–0.71	–0.86	46.5	45.8	–0.35	–0.45	49.5	54.0
P9	73	47.2	40.4	–0.57	–0.76	45.6	38.2	–0.46	–0.61	83.5	83.2
P10	30	36.6	35.1	–0.61	–1.14	37.6	35.2	–0.37	–0.74	60.2	62.6
P11	39	45.6	39.9	–0.59	–0.91	48.2	37.0	–0.47	–0.56	78.4	57.1
P12	8	48.8	37.1	–1.39	–1.20	48.2	36.0	–0.50	–0.49	35.1	40.6
P13	45	46.7	42.9	–0.89	–0.81	45.9	39.4	–0.45	–0.43	51.0	52.2
P14	–	–	–	–	–	–	–	–	–	–	–
P16	43	51.7	46.9	–0.55	–0.66	49.9	44.0	–0.36	–0.40	65.5	61.8
Mean ± SD	45 ± 31	45.1 ± 4.7	40.7 ± 4.7	–0.79 ± 0.25	-0.95 ± 0.18	44 ± 4.2	38.6 ± 4.2	–0.46 ± 0.08	–0.57 ± 0.12	62 ± 14	60 ± 11

**Fig. 10.**
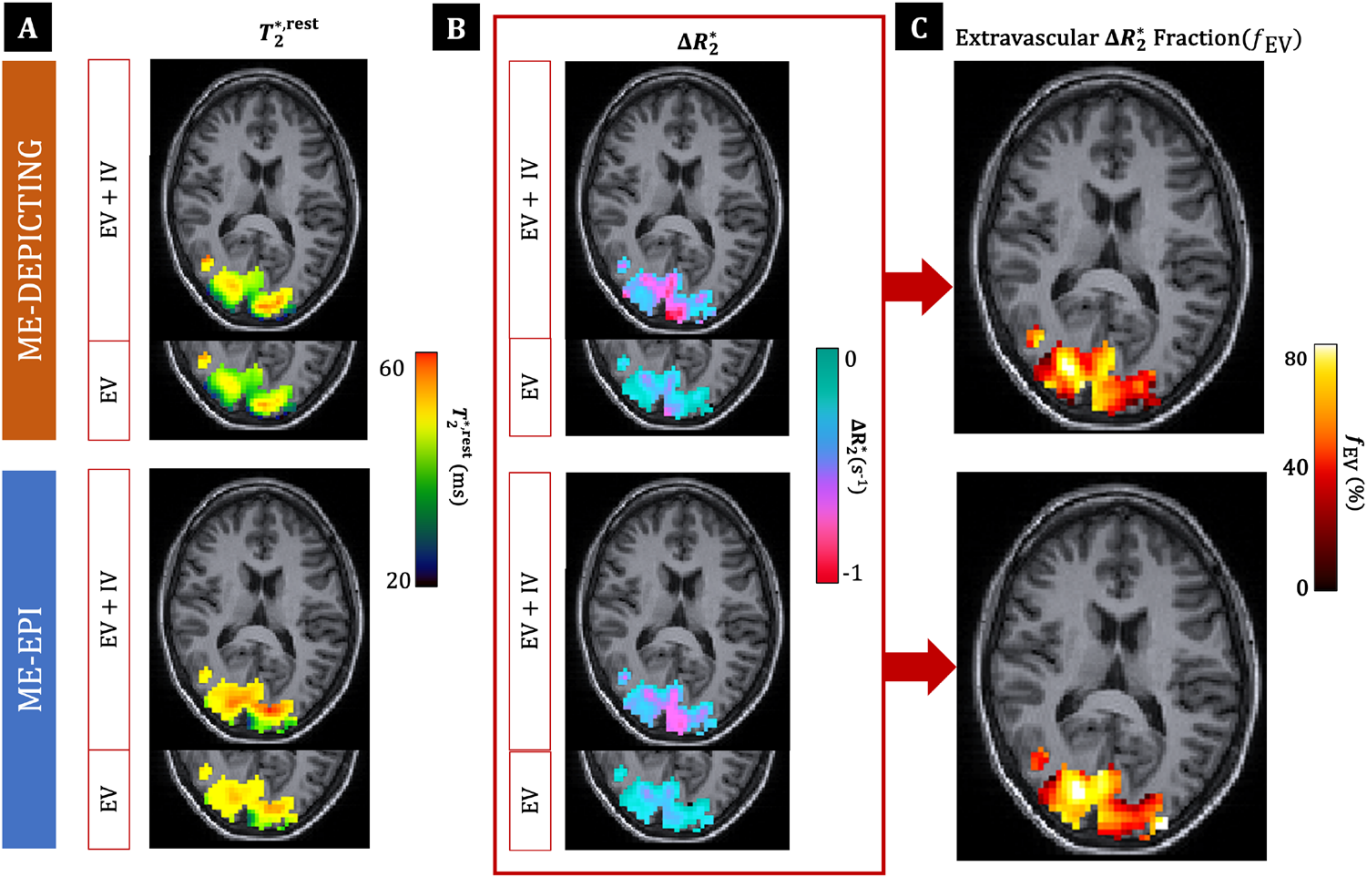
$T_{2}^{*,\text{rest}}$,$\Delta R_{2}^{*}$and$f_{\text{EV}}$maps for participant P16 (6th slice, common ROI-1). Voxel-wise results for (A)$T_{2,\text{EV+IV}}^{*,\text{rest}}$and$T_{2,\text{EV}}^{*,\text{rest}}$, (B)$\Delta R_{2,\text{EV}+\text{IV}}^{*}$and$\Delta R_{2,\text{EV}}^{*}$, and (C) the extravascular$\Delta R_{2}^{*}$fraction,$f_{\text{EV}}$obtained with ME-DEPICTING (top) and ME-EPI (bottom). The maps have been overlaid over the corresponding structural slice.

Increased estimates of the extravascular$\Delta R_{2}^{*}$fraction were obtained from the single-subject high-resolution DEPICTING data with$f_{\text{EV}}$= 80% (ROI-1, 458 voxels) and 95% (ROI-2, 237 voxels). A somewhat higher extravascular$\Delta R_{2}^{*}$fraction was also obtained with the 5-echo EPI data. These results along with those reported in recent literature for the visual cortex are summarized inTable 2.

**Table 2.** Summary of results from the EV+IV and EV compartments for$T_{2}^{*,\text{rest}}$and$\Delta R_{2}^{\text{*}}$, and the corresponding extravascular$\Delta R_{2}^{*}$fraction,$f_{\text{EV}}$, reported in literature for the visual cortex and from the current work.

Publication	Methodology	TE (ms)	Sample size	Voxel size (mm ^3^ )	ROI	EV+IV		EV		
						$T_{2}^{*,\text{rest}}$ (ms)	$\Delta R_{2}^{*}$ (s ^–1^ )	$T_{2}^{*,\text{rest}}$ (ms)	$\Delta R_{2}^{*}$ (s ^–1^ )	Extravasc. $\Delta R_{2}^{*}$ fraction $f_{\text{EV}}$ (%)
Lu and van Zijl (2005)	ME-VASO	14, 34.5, 55, 75.9	4	2 × 2 × 5	Single slice	45.5 ± 3.5	–0.58 ± 0.18	47.4 ± 3.1	–0.38 ± 0.10	67 ± 12
Donahue et al. (2011)	ME GRE-BOLD EPI with/without bipolar crushers	32.7, 44.6, 57.6, 70.7	7	3.5 × 3.5 × 3.5	Occipitallobe		–0.74 ± 0.13		–0.52 ± 0.19	70 ± 29
This work	SS-SI-VA	1.7, 10.7, 19.7	11	3 × 3 × 4	ROI-1	41.6 ± 3.7	–0.71 ± 0.16	39.0 ± 3.2	–0.38 ± 0.13	50 ± 12
	SO ME-DEPICTING				ROI-2	40.7 ± 4.7	–0.95 ± 0.18	38.6 ± 4.2	0.57 ± 0.12	60 ± 11
			1	1.7 × 1.7 × 1.7	ROI-1	47.5	–1.03	47.2	-0.80	80
					ROI-2	49.3	–1.13	47.2	-1.0	95
	SS-SI-VA	7.5, 20.7, 33.9	11	3 × 3 × 4	ROI-1	46.1 ± 3.9	–0.56 ± 0.13	45.0 ± 3.7	–0.31 ± 0.11	55 ± 15
	SO ME-EPI				ROI-2	45.1 ± 4.7	–0.79 ± 0.25	44.0 ± 4.2	–0.46 ± 0.08	62 ± 14
		10, 25.3, 40.6, 55.9, 71.2	1		ROI-1	43.1	–0.54	42.6	–0.31	59
					ROI-2	43.2	–0.59	42.8	–0.37	67

#### 3.3.2 Estimates of $\Delta {\text{R}}_{2,\text{IV}}^{\text{*}}$

Table 3lists the experimentally obtained data employed in the estimation of intravascular$\Delta R_{2,\text{IV}}^{*}$withEq. 4. Subject-averaged values have been provided for the multi-subject main study with 3-echoes and standard resolution. Values, corresponding to the participant (P1) from this study have also been provided along with their values for the single-subject study with 5 echoes (ME-EPI) and higher resolution (ME-DEPICTING). All values were based on ROI-2. The${\text{CBV}}^{\text{act}}$(${\text{CBV}}^{\text{rest}}+\Delta \text{CBV}$) values were obtained by assuming${\text{CBV}}^{\text{rest}}$= 0.05 ml/ml and are based on the$S_{\text{bn}}\left(\text{TE}\to 0\right)$data. Subject-averagedΔCBVvalues of 0.0053 ml/ml and 0.0067 ml/ml corresponded to percent CBV changes ofδcbv= 13 ± 3 and 11 ± 4% for EPI and DEPICTING, respectively. The difference between the results obtained with both readouts was insignificant (paired two-tailed*t*-test,p= 0.08). Similarly,δcbv= 11% was obtained for the 5-echo ME-EPI while the high-resolution DEPICTING data yielded the highestCBVchange,ΔCBV=0.0135 ml/ml orδcbv= 27%.

**Table 3.** List of experimental values used to estimate the intravascular$\Delta R_{2,\text{IV}}^{*}$for the two readouts based on the results in ROI-2.$\delta s_{\text{VASO}}$values were extracted from the dynamically divided data at the respective echo time.

	TE _mid_ (ms)	${\text{CBV}}^{\text{act}}$ (ml/ml)	$\delta s_{\text{VASO,dd}}\left({\text{TE}}_{\text{mid}}\right)$ (%)	$T_{2,\text{EV}}^{*,\text{rest}}$ (ms)	$T_{2,\text{EV}}^{*,\text{act}}$ (ms)
ME-DEPICTING					
11 subjects, averaged	10.7	0.0553	–0.9	38.6	39.5
P1 (3 × 3 × 4 mm ^3^ )	10.7	0.0573	–1.1	31.4	32.1
P1 (1.7 × 1.7 × 1.7 mm ^3^ )	15.1	0.0635	–1.0	49.0	51.5
ME-EPI					
11 subjects, averaged	20.7	0.0567	–1.5	44.4	45.3
P1 (3 echoes)	20.7	0.0579	–1.7	41.4	42.3
P1 (5 echoes)	40.6	0.0553	–1.3	42.8	43.5

**Fig. 11.**
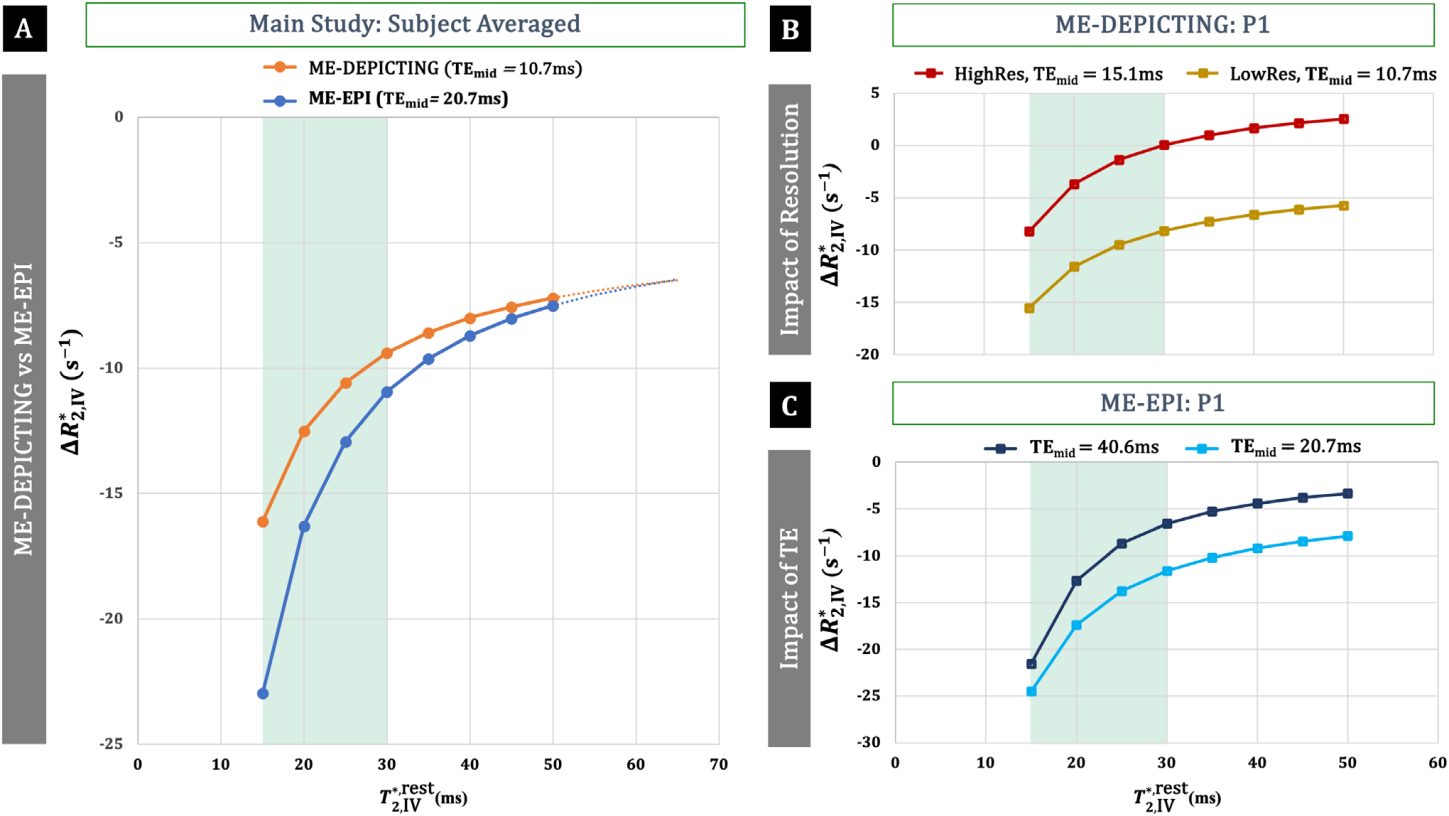
Dependence of$\Delta R_{2,\text{IV}}^{*}$on$T_{2,\text{IV}}^{*,\text{rest}}$. (A) Results obtained with subject-averaged input values for ME-DEPICTING (orange) and ME-EPI (blue). (B) High-resolution versus low-resolution data obtained in a single participant (P1) with ME-DEPICTING. (C) Single-subject results (P1) obtained with ME-EPI at different TE. The shaded green area represents the venous range with$T_{2,\text{IV}}^{*,\text{rest}}$≈ 15–30 ms (Donahue et al., 2011).

The results for the intravascular$\Delta R_{2,\text{IV}}^{*}$estimations are plotted inFigure 11for assumed$T_{2,\text{IV}}^{*,\text{rest}}$values ranging from 15 to 50 ms. A nonlinear relationship between$\Delta R_{2,\text{IV}}^{*}$and$T_{2,\text{IV}}^{*,\text{rest}}$is evidenced in all the plots. A steeper decline of$\left|\Delta R_{2,\text{IV}}^{*}\right|$is observed in the region of venous$T_{2,\text{IV}}^{*,\text{rest}}$. The differing$\Delta R_{2,\text{IV}}^{*}$contributions between the two readouts can also be seen converging at higher resting$T_{2}^{*}$values (Fig. 11A). The within-sequence comparisons demonstrate a lower intravascular contribution (i.e., smaller$\left|\Delta R_{2,\text{IV}}^{*}\right|$) with higher spatial resolution (Fig. 11B) and longerTE(Fig. 11C).

## 4 Discussion

Readouts with short TE come with the promise of better sensitivity for non-BOLD contrasts (Hetzer et al., 2011;Huber et al., 2019) as BOLD contamination atTE<2 ms is expected to be minimal. Our recent pCASL study at 3 T had proven this to be the case for functional CBF changes in the visual cortex (Devi et al., 2022). The area of activation obtained with pCASL-prepared ME-DEPICTING at${\text{TE}}_{1}$= 1.7 ms exceeded that obtained with ME-EPI at${\text{TE}}_{1}$= 8 ms by 40%. The present study confirms the same for the VASO contrast. The sensitivity for detecting uncorrected VASO signal changes at${\text{TE}}_{1}$of the blood-nulled dataset,$S_{\text{bn}}\left({\text{TE}}_{1}=1.7\text{ ms}\right)$was found to be substantially higher than that of ME-EPI,$S_{\text{bn}}\left({\text{TE}}_{1}=7.5\text{ ms}\right)$. However, comparing the functional sensitivity of data BOLD-corrected by ME extrapolation toTE=0(Fig. 3B), an almost equivalent VASO CNR was obtained for both readouts. This differs from the results of the pCASL study, wherein a higher CBF-CNR was obtained for the ME-DEPICTING acquisitions extrapolated to zeroTE[Fig. 6C and 6E inDevi et al. (2022)] with approximately the same TEs as employed in the current work. Also contrary to the VASO results was a higher CNR for CBF changes obtained with${\text{TE}}_{1}$data for both readouts compared to the corresponding$S_{0}$data. An explanation for the improvement of the quality of the fitted data atTE→0in SS-SI-VASO could be the fact that the data were first split into blood-nulling and control images prior to extrapolation, whereas the pCASL data were fit as a whole, with the fluctuations between the control and label data, possibly degrading the quality of the resulting$S_{0}$estimate. Nonetheless, as with the pCASL study, the sensitivity for the BOLD response obtained from the$R_{2,\text{ctr}}^{*}$and$S_{\text{ctr}}\left(\text{Sum}\right)$data was found to be comparable for both readouts (Fig. 3CandSupplementary Table S4). For instance,$R_{2,\text{ctr}}^{*}$yields 1500 ± 448 suprathreshold voxels with ME-DEPICTING vs. 1420 ± 439 voxels with ME-EPI (p= 0.60; CNR 3.2±0.9 vs. 3.7±1.1,p= 0.05). This finding, along with the prediction of a shorter${\text{TE}}_{1}$providing a better approximation of CBV (Genois et al., 2021), makes ME-DEPICTING a promising substitute of the traditional EPI readout for the simultaneous measurement of CBV changes accompanying the BOLD response.

### 4.1 BOLD correction by dynamic division

A much higher gain in VASO sensitivity was deduced for the${\text{TE}}_{1}$data corrected for BOLD contributions by dynamic division compared to the correction by extrapolation to zero TE for both readouts. Further investigation into this remarkable improvement revealed a linear TE dependence of the BOLD-corrected$\delta s_{\text{VASO,dd}}$data (Figs. 6A,6D,7Aand7C). This TE dependence bears a close resemblance to that of the BOLD data, the difference being that the latter’s intercept is close to zero (Figs. 6C,6F,7Band7D). This suggests a failure of the dynamic division strategy and, hence, the presence of residual BOLD contamination in the$\delta s_{\text{VASO,dd}}$data, as also hinted by the similar but mirrored shape of their time courses. The VASO measures obtained from extrapolated and even uncorrected data followed temporal dynamics expected for a functional CBV response: a slower return to baseline and less prominent post-stimulus transients (Lu et al., 2003;Mandeville et al., 1998). Dynamic division-corrected data, on the other hand, showed an earlier return to baseline and post-stimulus overshoots that increased in peak amplitude with TE (Figs. 5and8B). This finding mimics that of the BOLD response in ME acquisitions (Havlicek et al., 2017) and was even more evident in the ME-EPI data with extended echo train length (${\text{TE}}_{5}$= 71.2 ms) (Fig. 8B).

#### 4.1.1 Intravascular BOLD contribution

Relating VASO signal changes to the CBV response in SS-SI-VASO (Eq. 1) relies on the assumption of equivalent BOLD contributions in the blood-nulling and control condition (Huber, Ivanov, et al., 2014). This assumption holds in situations where the BOLD signal is mostly of extravascular origin. The extent of intravascular BOLD contributions to the BOLD signal, however, relies on a number of factors, including field strength ($B_{0}$), TE, readout sequence, and diffusion weighting (Duong et al., 2003;Silvennoinen et al., 2003;Uludaǧ et al., 2009). The intravascular$\Delta R_{2}^{*}$fraction is expected to decrease with$B_{0}$with predicted intravascular BOLD contributions from the microvasculature of approximately 57%, 36%, 11%, and 5% at the respective field strengths of 1.5 T, 3 T, 4 T, and 4.7 T andTE=$T_{2,\text{EV}}^{*}$(Uludaǧ et al., 2009). Experimentally obtained extravascular$\Delta R_{2}^{*}$fractions are approximately 70% at 3 T (Donahue et al., 2011;Lu et al., 2003) and approximately 90% at 7 T (Cheng et al., 2015;Donahue et al., 2011) in the human visual cortex. The results from the current study are well within the range of what has been reported (Table 2). Our estimated$\Delta R_{2,\text{b}}^{*}$values (Fig. 11) agree also with previous*in-vitro*measurements in bovine blood of varying hematocrit (Hct) levels ($\Delta R_{2,\text{b}}^{*}$= –8.16 s^–1^,—14.3 s^–1^, and—16.6 s^–1^for Hct = 0.21, 0.44, and 0.57, respectively), with the assumption of changes in blood oxygen saturation fraction,*Y*, from 0.61 to 0.73 (Zhao et al., 2007).

The observed linear dependence onTEof the VASO data corrected by dynamic division align with simulations based on a vascular anatomical model (Genois et al., 2021), wherein the intravascular BOLD contributions were taken into account. The linear dependence of$\delta s_{\text{VASO,dd}}$onTEranging from 0 to 30 ms as depicted inFigure 5AofGenois et al. (2021)for 3-T data with an extravascular fraction of 72% resemble our plots inFigure 6Aand6D.

Our results, however, differ from those reported in the original SS-SI-VASO paper (Huber, Ivanov, et al., 2014). The 3-T results therein, with data from two participants, found$\delta s_{\text{VASO,dd}}$to be independent of TE (14 ms and 30 ms) in the visual cortex. This is remarkable as at a nominal resolution of 3 × 3 × 4 mm^3^, partial voluming effects are expected to be at play and introduce significant intravascular BOLD contributions. For instance, with the assumption of a 5% increase in oxygen metabolism, 57.5% increase in CBF, TE = 50 ms, and TR = 1s,Zhao et al. (2007)estimated an intravascular BOLD contribution of 13% from pure parenchyma, which increased to a substantial 42% for parenchyma contaminated by 2% veins. Consistently, a reduction in intravascular BOLD contributions from larger veins at higher resolution is evident in our single-subject data (Fig. 11B). Interestingly, despite an estimated 80–90% extravascular$\Delta R_{2}^{*}$fraction for this data, the intravascular fraction was sufficient to bring about a TE dependence of$\delta s_{\text{VASO,dd}}$.

A TE independence of 3-T ME-EPI data (12 ms and 48 ms) corrected by dynamic division has been reported in a recent layer-fMRI VASO study at sub-millimeter resolution (Huber, Kronbichler, et al., 2023), where contamination by larger veins is expected to be largely reduced. It should be noted that even in the absence of intravascular BOLD signal, any CBV change leads to a TE-dependent SS-SI-VASO signal induced by the TE dependence of the resting signals of corresponding (arterial or venous) blood compartments relative to the tissue signal. Consequently, an SS-SI-VASO signal increasing withTEwas also found at 7 T in a study employing a resolution of 1.5 mm (Fig. 5AinHuber, Goense, et al., 2014). Quantification of CBV changes during activation atTE>0will always be governed by such biases. Under certain circumstances, however, these biases might cancel out forTE>0, because such arterial and venous contributions tend to increase and decrease the SS-SI-VASO signal, respectively.

#### 4.1.2 Relationship between SS-SI-VASO signals

The echo-wise evaluation indicated a remarkable similarity of amplitude and transients of$\delta s_{\text{VASO,dd}}$and the difference signal, defined as$\delta s_{\text{VASO,bn}}-\delta s_{\text{BOLD,ctr}}$(Fig. 5). Differences in the extravascular$\Delta R_{2}^{*}$fraction and, subsequently, in intravascular BOLD contaminations do not explain this finding. As shown inAppendix A2, the similarity of both quantities is easily explained by the relation$\delta s_{\text{VASO,dd}}=\left(S_{\text{ctr}}^{\text{rest}}/S_{\text{ctr}}^{\text{act}}\right)\left(\delta s_{\text{VASO,bn}}-\delta s_{\text{BOLD,ctr}}\right)$(seeEq. A9). Because$S_{\text{ctr}}^{\text{rest}}\leq S_{\text{ctr}}^{\text{act}}$holds (positive BOLD response) and given the small stimulus-related signal change (~1–2%),$\left|\delta s_{\text{VASO,dd}}\right|$is expected to be slightly smaller than$\left|\delta s_{\text{VASO,bn}}-\delta s_{\text{BOLD,ctr}}\right|$. This relationship, however, could explain the stabilization of$\delta s_{\text{VASO,dd}}$at longerTEin our 5-echo example. Since$\delta s_{\text{BOLD,ctr}}$contains an intravascular signal whereas$\delta s_{\text{VASO,bn}}$does not, their difference is expected to vary faster at shorterTE, because at 3 T, the intravascular signal increases significantly more than the extravascular (Triantafyllou et al., 2011).

#### 4.1.3 Imaging sequence-related effects

Differences between the EPI readouts employed in previous studies and the DEPICTING readout, such as different$T_{2}^{*}$-sensitivities of the specific k-space trajectories (Devi et al., 2022;Patzig et al., 2021), may contribute to the failure of the dynamic-division strategy in the present work. Although this aspect is likely to be of only secondary order because a TE dependence was introduced in our$\delta s_{\text{VASO,dd}}$data and correspondingδcbfacquired with EPI as well (Fig. 6A,6B,6Dand6E), it cannot be ignored. As shown inAppendix A3, additional$T_{2}^{*}$-weighting due to the k-space trajectory would result in non-zero$\delta s_{\text{ctr}}\left(\text{TE}\to 0\right)$and may be captured by an offset term${\text{TE}}_{t}$leading to an effective echo time,${\text{TE}}_{\text{eff}}=\text{TE}+{\text{TE}}_{t}$. With the intercepts obtained inFigure 7Band7Dand the results fromTable 2,Eq. A11yields estimates of${\text{TE}}_{t}$≈ 0.96 ms and 0.35 ms for DEPICTING and EPI, respectively. The larger offset${\text{TE}}_{t}$for DEPICTING is due to the different$T_{2}^{*}$-sensitivity of its k-space trajectory (Devi et al., 2022;Patzig et al., 2021). A significant deviation from the nominal TE results only at${\text{TE}}_{1}$of DEPICTING (${\text{TE}}_{t}$≈ 0.56${\text{TE}}_{1}$), but no relevant bias at longer TEs and for EPI. This effect leads to the slightly lowerδcbvvalue estimated from$S_{\text{bn}}\left(\text{TE}\to 0\right)$of DEPICTING (11 ± 4%) compared to that of EPI (13 ± 3%) and also explains somewhat larger BOLD-like fluctuations in its extrapolated$\delta s_{\text{ctr}}$signal (cf., participants P11 and P12 inSupplementary Fig. S2). The different zero-crossing TE of the$\delta s_{\text{bn}}$of the two readouts (Supplementary Fig. S3: approximately 11 ms and 17 ms for DEPICTING and EPI, respectively) could also be attributed to this difference in BOLD weighting at equivalent TEs.

A distinction lies in the TEs employed by earlier studies, which were comparatively longer and, hence, less likely to be significantly influenced by intravascular BOLD contributions. A consistent tendency could be the near-plateauing of$\delta s_{\text{VASO,dd}}$values at longer TE of the ME-EPI data, while the ME-DEPICTING data with shorter TEs appear to follow an increasing trend over the entire range (Fig. 6Aand6D). This is also evident in time courses of later echoes extracted from the nulled slice of ME-EPI inFigures 5and9B. Further ME studies might provide additional insight into this matter. Such studies are, however, presently rare, even with SS-SI-VASO being the current workhorse of CBV-based layer-fMRI studies. Apart from the work mentioned above, all other studies used single-echo acquisitions at 7 T with 15 ms ≤TE≤ 28 ms (Beckett et al., 2020;de Oliveira et al., 2023;Faes et al., 2023;Guidi et al., 2016,^2020^;Huber et al., 2015,^2021^;Huber, Kassavetis, et al., 2023;Liu et al., 2022;Oliveira et al., 2021,^2022^;Pizzuti et al., 2023) and with 27 ms at 3 T (Knudsen et al., 2023). We note that we do not expect relevant differences between the 2D readouts used here and those of the 2D Simultaneous Multi-Slice (SMS) EPI and 3D EPI variants (Huber et al., 2018), given that evaluations were exclusively based on the nulled slice.

Short TEs can also be achieved with spiral acquisitions. With recent developments in their implementation, artifact correction, and reconstruction, these can be expected to be a promising alternative to EPI sequences with Cartesian trajectories for non-BOLD fMRI (Glover, 2012;Kasper et al., 2022). A preliminary report of a two-fold improvement in temporal SNR (tSNR) compared to 3D-EPI was recently presented for VASO fMRI with spiral readouts at 7 T (Monreal-Madrigal et al., 2023).

### 4.2 Limitations

SS-SI-VASO was implemented in the present study for TI/TR = 1153 ms/2000 ms. This fulfills the requirements of the original paper [see Fig. 1a ofHuber, Ivanov, et al. (2014)] by choosing a TI shorter than the arterial transit time of blood and a ‘period III’ (i.e.,2TR+TI) that prevents contamination with blood that was inverted more than once. Our timing, however, does not null the CSF contribution to the signal. A higher extravascular signal intensity was preferred over CSF nulling by our choice of a shorter TR compared to that required for simultaneous nulling of blood and CSF contributions (TR = 2.75 s). Pilot experiments had revealed a more focal activation with CSF nulling. However,$\delta s_{\text{VASO},\text{bn}}\left(\text{TE}\to 0\right)$values within a common ROI were found to be very similar. Nonetheless, partial volume effects due to CSF contribution cannot be disregarded in our results. The TI of 1153 ms was based on a blood$T_{1}$value of 1664 ms measured from bovine blood at 3 T (Lu, Clingman, et al., 2004), which is lower than recently published$T_{1}$values (~1800 ms) of human blood (Li et al., 2016,^2017^). With our approximate slice acquisition time of 71 ms, our choice of a TI might has nulled the 7th rather than the 6th slice. This is expected to result in only minimal blood contributions and, hence in significant over- or underestimations ofCBV changes.

The ROI definition in the current study could also have been attributed to deviations from the original report. The voxel-based thresholding employed in the present work could bias the selection to voxels that showed the maximum percent signal changes and, hence, towards voxels with more partial voluming with blood vessels. On the other hand, the more widely employed cluster-based thresholding is sensitive to weaker and more diffuse signal changes, while suffering from lower spatial specificity (Woo et al., 2014).

The very short TE achieved with ME-DEPICTING was of primary interest for the current work and as such, 2D readouts were compared. While the error due to incompletely nulled slices was corrected for evaluatingΔCBV, such corrections are not easily obtained for$\Delta R_{2}^{*}$values. Consequently, estimations of$\Delta R_{2,\text{EV}}^{*}$, and hence also$\Delta R_{2,\text{IV}}^{*}$, were limited to the single blood-nulled slice. Future work with 2D SMS-EPI (Huber et al., 2016) and SMS-DEPICTING or corresponding 3D readouts could solve this issue. At 7 T, 2D SMS-EPI was found to outperform 3D EPI (Poser et al., 2010) at lower resolutions due to lesser contributions from physiological noise (Huber et al., 2018).

Only a single pilot acquisition at higher resolution, which is likely to be less biased by intravascular BOLD contributions, and another one with more echoes have been provided here. Increasing the number of participants in each could further solidify these findings. Assessing the TE dependence may benefit from a further increase of the number of echoes. Extending the evaluation beyond the visual system, such as the motor cortex, is also warranted. The impact of intravascular BOLD contributions might also be investigated by combining 3-T experiments with those at 7 T, where the extravascular BOLD effect is expected to be dominant. Further experimentation is, indeed, needed to comment on suitable ranges ofTEand spatial resolutions, within which the dynamic division method would be feasible at the respective field strengths. This would help identify the impact of the dynamic-division correction in SS-SI-VASO-based layer fMRI studies.

Presently, in the absence of a ground truth,$\delta s_{\text{VASO},\text{bn}}\left(\text{TE}\to 0\right)$and, subsequently,δcbvvalues obtained by extrapolation to zero TE appear to deliver the most reliable estimations, especially, when quantification is the goal. However, the assumed mono-exponential$T_{2}^{*}$decay with TE is likely to be too simplistic and may introduce some bias.

## 5 Conclusion

SS-SI-VASO was implemented at 3 T with two 2D readouts. The feasibility of the ME-DEPICTING sequence as a potentially advantageous implementation compared to ME-EPI was investigated, motivated by the shorter${\text{TE}}_{1}$and inter-echo time capabilities of this double-shot EPI readout. Unwanted BOLD contamination was, in fact, drastically reduced at the very short${\text{TE}}_{1}$of 1.7 ms. The extrapolation to zero TE, however, resulted in equivalent VASO sensitivity for the two readouts. Interestingly, the dynamic division approach was found to bring about a higher gain in VASO sensitivity for ME-EPI than for ME-DEPICTING. VASO signal changes corrected for BOLD with this strategy were, however, found to exhibit a distinctTE- dependence. This is probably due to the influence of intravascular BOLD contributions at 3 T. The correction for BOLD contamination by extrapolation to zero TE using multiple echoes does not rely on a negligible intravascular BOLD signal and is, therefore, recommended, if an overestimation of functional CBV changes is to be avoided. Consequently, in the absence of ME data, the shortTEof the DEPICTING readout still provides an alternative to conventional EPI if better sensitivity and accuracy of the VASO data are desired.

## Supplementary Material

Supplementary Material

## Data Availability

Pre-processed data for all echoes, data derived from multi-echo fitting and relevant scripts for BOLD correction by dynamic division and estimation of$\Delta R_{2,\text{IV}}^{*}$are available athttps://osf.io/cg7sp/.

## Footnotes

1 ‘*S*’ denotes a certain type of data or time series as indicated by an additional subscript (‘_bn_’ for blood-nulled, ‘_ctr_’ for control, or ‘_dd_’ for dynamically divided). The corresponding TE is further specified in parentheses, for example,$S_{\text{bn}}\left({\text{TE}}_{i}\right)$,$S_{\text{ctr}}\left({\text{TE}}_{i}\right)$or$S_{\text{dd}}\left({\text{TE}}_{i}\right)$, whereiindicates the echo number. Data obtained by extrapolation to zero TE or a weighted summation of data acquired at multiple TEs are denoted as ‘S(TE→0)’ and ‘S(sum)’, respectively. For simplicity, ME datasets acquired during the blood-nulled and control conditions are sometimes referred to as ‘VASO data’ and ‘BOLD data’, respectively. The relative signal change expressed as percent of the resting value is denoted as ‘δs’ plus a subscript indicating the contrast (‘_VASO_’ or ‘_BOLD_’) and the data type (as above) as well as the TE information. Percent signal changes between activation and rest are therefore written as$\delta s_{\text{VASO,bn}}\left({\text{TE}}_{i}\right)$,$\delta s_{\text{BOLD,ctr}}\left({\text{TE}}_{i}\right)$or$\delta s_{\text{VASO,dd}}\left({\text{TE}}_{i}\right)$. A comprehensive list of the notations is given in the Supplementary Material.

## Appendix A1. Intravascular BOLD Response

The signal intensities recorded during the blood-nulling (bn) and control (ctr) conditions in a parenchyma (EV+IV) voxel containing extravascular tissue and an intravascular compartment (blood) can be written as (Huber, 2015;Huber, Goense, et al., 2014;Huber, Ivanov, et al., 2014), respectively

$$S_{\text{bn}}\propto V_{\text{EV}}\rho_{\text{EV}}M_{0}AE_{2,\text{EV}}$$
(A1)

and

$$S_{\text{ctr}}\propto V_{\text{EV}}\rho_{\text{EV}}M_{0}BE_{2,\text{EV}}+\text{CBV}\rho_{\text{IV}}M_{0}CE_{2,\text{IV}}$$
(A2)

with$A\equiv 1-2\exp\left(-\text{TI}/T_{1,\text{EV}}\right)+\exp\left(-\text{TR}/T_{1,\text{EV}}\right)$,$B\equiv 1-\exp\left(-\text{TR}/T_{1,\text{EV}}\right)$,$C\equiv 1-\exp\left(-\text{TR}/T_{1,\text{IV}}\right)$, and$E_{2,i}\equiv \exp\left(-\text{TE}/T_{2,i}^{*}\right)$withi∈{EV+IV, EV,IV}.$V_{i}$and$\rho_{i}$are the volumes and the relative proton densities (compared to water), and$M_{0}$is the equilibrium magnetization. Due to the preservation of mass,$V_{\text{EV+IV}}\cdot \rho_{\text{EV+IV}}=V_{\text{EV}}\cdot \rho_{\text{EV}}+\text{CBV}\cdot \rho_{\text{IV}}$, and the absolute activation-related VASO signal change can be written as:

$$S_{\text{VASO,dd}}=\frac{S_{\text{bn}}^{\text{act}}}{S_{\text{ctr}}^{\text{act}}}-\frac{S_{\text{bn}}^{\text{rest}}}{S_{\text{ctr}}^{\text{rest}}}=\frac{A\beta^{\text{act}}}{B\beta^{\text{act}}+C\alpha^{\text{act}}\frac{E_{2,\text{IV}}^{\text{act}}}{E_{2,\text{EV}}^{\text{act}}}}-\frac{A\beta^{\text{rest}}}{B\beta^{\text{rest}}+C\alpha^{\text{rest}}\frac{E_{2,\text{IV}}^{\text{rest}}}{E_{2,\text{EV}}^{\text{rest}}}}$$
(A3)

with$\alpha^{j}\equiv {\text{CBV}}^{j}\cdot \rho_{\text{IV}}/V_{\text{EV+IV}}$,$\beta^{j}\equiv \left(\rho_{\text{EV+IV}}-\alpha^{j}\right)$andj∈{act,rest}, such that

$$\delta s_{\text{VASO,dd}}=\frac{\Delta S_{\text{VASO,dd}}}{\frac{S_{\text{bn}}^{\text{rest}}}{S_{\text{ctr}}^{\text{rest}}}}\times 100\%=\left(\frac{\beta^{\text{act}}}{\beta^{\text{rest}}}\times \frac{B\beta^{\text{rest}}+C\alpha^{\text{rest}}\frac{E_{2,\text{IV}}^{\text{rest}}}{E_{2,\text{EV}}^{\text{rest}}}}{B\beta^{\text{act}}+C\alpha^{\text{act}}\frac{E_{2,\text{IV}}^{\text{act}}}{E_{2,\text{EV}}^{\text{act}}}}-1\right)\times 100\%$$
(A4)

and

$$\delta s_{\text{VASO}}=\left(\frac{B+C\frac{\alpha^{\text{rest}}}{\beta^{\text{rest}}}\cdot \frac{E_{2,\text{IV}}^{\text{rest}}}{E_{2,\text{EV}}^{\text{rest}}}}{B+C\frac{\alpha^{\text{act}}}{\beta^{\text{act}}}\cdot \frac{E_{2,\text{IV}}^{\text{act}}}{E_{2,\text{EV}}^{\text{act}}}}-1\right)\times 100\%.$$
(A5)

Equation A4permits an estimation of the intravascular BOLD effect (i.e.,$\Delta R_{2,\text{IV}}^{*}$), as a function of$E_{2,\text{IV}}^{\text{rest}}$, from

$$E_{2,\text{IV}}^{\text{act}}=E_{2,\text{EV}}^{\text{act}}\frac{\frac{\alpha^{\text{rest}}E_{2,\text{IV}}^{\text{rest}}}{\beta^{\text{rest}}E_{2,\text{EV}}^{\text{rest}}}-\frac{\delta s_{\text{VASO,dd}}}{100\%}\cdot \frac{B}{C}}{\frac{\alpha^{\text{act}}}{\beta^{\text{act}}}\left(\frac{\delta s_{\text{VASO,dd}}}{100\%}+1\right)}.$$
(A6)

## Appendix A2. Relationship Between SS-SI-VASO Signals

The activation-related VASO-signal change with dynamic division is given by:

$$\begin{array}{l} \delta s_{\text{VASO,dd}}=\frac{\frac{S_{\text{bn}}^{\text{act}}}{S_{\text{ctr}}^{\text{act}}}-\frac{S_{\text{bn}}^{\text{rest}}}{S_{\text{ctr}}^{\text{rest}}}}{\frac{S_{\text{bn}}^{\text{rest}}}{S_{\text{ctr}}^{\text{rest}}}}\times 100\%\\ =\frac{S_{\text{bn}}^{\text{act}}S_{\text{ctr}}^{\text{rest}}-S_{\text{bn}}^{\text{rest}}S_{\text{ctr}}^{\text{act}}}{S_{\text{bn}}^{\text{rest}}S_{\text{ctr}}^{\text{act}}}\times 100\%, \end{array}$$
(A7)

whereas the difference between the uncorrected signal change in the nulling condition and the signal change in the control condition is given by:

$$\begin{array}{l} \delta s_{\text{VASO,bn}}-\delta s_{\text{BOLD,ctr}}=\left(\frac{S_{\text{bn}}^{\text{act}}-S_{\text{bn}}^{\text{rest}}}{S_{\text{bn}}^{\text{rest}}}-\frac{S_{\text{ctr}}^{\text{act}}-S_{\text{ctr}}^{\text{rest}}}{S_{\text{ctr}}^{\text{rest}}}\right)\times 100\%\\ =\frac{S_{\text{bn}}^{\text{act}}S_{\text{ctr}}^{\text{rest}}-S_{\text{bn}}^{\text{rest}}S_{\text{ctr}}^{\text{act}}}{S_{\text{bn}}^{\text{rest}}S_{\text{ctr}}^{\text{rest}}}\times 100\%. \end{array}$$
(A8)

This leads to a ratio of the two quantities according to:

$$\frac{\delta s_{\text{VASO,dd}}}{\delta s_{\text{VASO,bn}}-\delta s_{\text{BOLD,ctr}}}=\frac{S_{\text{ctr}}^{\text{rest}}}{S_{\text{ctr}}^{\text{act}}}.$$
(A9)

## Appendix A3. Effective TE of the Readout Sequence

With both EPI and DEPICTING, each k-space line is acquired at an individual echo time, so the effective$T_{2}^{*}$-weighting may differ from the first-order approximation based on the TE of the central k-space line. To analyze the influence from the readout trajectory, we define an effective echo time,${\text{TE}}_{\text{eff}}=\text{TE}+{\text{TE}}_{t}$, that considers an additional term${\text{TE}}_{t}$due to the readout trajectory. Consideration of${\text{TE}}_{t}$and a first-order Taylor expansion of$\exp\left(-\text{TE}\times R_{2}^{*}\right)$, the BOLD-weighted signal change atTE =0during the control condition is, therefore, given by

$$\begin{array}{l} \delta s_{\text{ctr}}\left(\text{TE}\to 0\right)=\frac{S_{\text{ctr}}^{\text{act}}-S_{\text{ctr}}^{\text{rest}}}{S_{\text{ctr}}^{\text{rest}}}\times 100\%\\ \approx \frac{-{\text{TE}}_{t}\left(R_{2,\text{EV+IV}}^{*,\text{act}}-R_{2,\text{EV+IV}}^{*,\text{rest}}\right)}{1-{\text{TE}}_{t}R_{2,\text{EV+IV}}^{*,\text{rest}}}\times 100\%, \end{array}$$
(A10)

which leads to

$${\text{TE}}_{t}\approx \frac{\delta s_{\text{ctr}}\left(\text{TE}\to 0\right)/100\%}{\frac{\delta s_{\text{ctr}}\left(\text{TE}\to 0\right)/100\%}{T_{2,\text{EV+IV}}^{*,\text{rest}}}-\Delta R_{2,\text{EV}+\text{IV}}^{*}}.$$
(A11)
