# Supplementary material for: A comparative analysis of the sensitivity and BOLD contamination of the VASO response at 3 Tesla: ME-DEPICTING vs. ME-EPI readouts
Source: Imaging Neurosci (Camb). 2024 Oct 28;2:imag-2-00333. doi: 10.1162/imag_a_00333 (PMC12290853; doi:10.1162/imag_a_00333)
Supplement: Supplementary Material [file imag_a_00333-supp.pdf]

Supplementary Material

A Comparative Analysis of the Sensitivity and BOLD contamination of the VASO response at 3 Tesla: ME-DEPICTING vs ME-EPI readouts

Ratnamanjuri Devi[1], Jöran Lepsien[1], Toralf Mildner[1], Harald E. Möller[1]

[1]NMR Methods & Development Group, Max Planck Institute for Human Cognitive and Brain Sciences, Leipzig, Germany

Notations

BOLD:	Blood Oxygenation Level Dependent,
CBF:	Cerebral blood flow in units of ml blood per 100g of tissue per minute,
CBV:	Cerebral blood volume in units of ml blood per ml of tissue,
$\mathrm{CBV}^{\mathrm{act}}$:	Cerebral blood volume in the activated state,
$\mathrm{CBV}^{\mathrm{rest}}$ or V_{IV}:	Cerebral blood volume in the resting state,
δcbv:	Estimated % CBV change as obtained from δs_{VASO} by assuming a $\mathrm{CBV}^{\mathrm{rest}}$ of 0.05 ml/ml,
ΔCBV:	Estimated absolute CBV change as obtained from δs_{VASO} and corrected for imperfect slice nulling,
EV:	Extravascular,
f_{EV}:	EV ΔR_2^* fraction in %,
i:	Integer denoting the echo number; i = 1,2,3, …,
IV:	Intravascular,
M_0:	Equilibrium magnetization,
$R_{2,\mathrm{bn}}^*$	R_2^* image obtained from fitting all echoes of the blood-nulled data, $S_{\mathrm{bn}}(\mathrm{TE}_i)$,
$R_{2,\mathrm{ctr}}^*$	R_2^* image obtained from fitting all echoes of the control data, $S_{\mathrm{ctr}}(\mathrm{TE}_i)$,
$\Delta R_{2,\mathrm{bn}}^*$:	Absolute change in R_2^* obtained from $R_{2,\mathrm{bn}}^*$ between activation and rest in s^{-1},
$\Delta R_{2,\mathrm{ctr}}^*$:	Absolute change in R_2^* obtained from $R_{2,\mathrm{ctr}}^*$ between activation and rest in s^{-1},
$\Delta R_{2,\mathrm{EV}}^*$:	ΔR_2^* of tissue i.e from EV compartment in s^{-1}; estimated from the nulled slice of $R_{2,\mathrm{bn}}^*$,
$\Delta R_{2,\mathrm{EV+IV}}^*$:	Different name for $\Delta R_{2,\mathrm{ctr}}^*$, to clarify the difference to $\Delta R_{2,\mathrm{EV}}^*$ as it is influenced by both EV and IV compartments,
$\Delta R_{2,\mathrm{IV}}^*$:	ΔR_2^* of blood i.e from IV compartment in s^{-1}; estimated by a VASO signal model,
ρ_{EV}:	Water proton density of the EV compartment, in ml water/ml gray matter,
$\rho_{\mathrm{EV+IV}}$:	Water proton density of the parenchyma (EV+IV), in ml water/ml parenchyma,
ρ_{IV}:	Water proton density of the IV compartment, in ml water/ml blood,

S:	Image, data or signal,
$S(\mathrm{TE}\rightarrow 0)$:	Multi-echo (ME) data extrapolated to TE=0,
$S_{\mathrm{bn}}(\mathrm{TE}\rightarrow 0)$:	$S(\mathrm{TE}\rightarrow 0)$ image obtained from ME extrapolation of the blood-nulled images, $S_{\mathrm{bn}}(\mathrm{TE}_i)$, to TE→0,
$S_{\mathrm{bn}}(\mathrm{TE}_i)$:	Blood-nulled (VASO) dataset of echo i,
$S_{\mathrm{ctr}}(\mathrm{sum})$:	Combined ME dataset obtained from a weighted summation of $\mathrm{S}_{\mathrm{ctr}}(\mathrm{TE}_{\mathrm{i}})$,
$S_{\mathrm{ctr}}(\mathrm{TE}\rightarrow 0)$:	$S(\mathrm{TE}\rightarrow 0)$ image obtained from ME extrapolation of the control images, $S_{\mathrm{ctr}}(\mathrm{TE}_i)$ to TE→0,
$S_{\mathrm{ctr}}(\mathrm{TE}_i)$:	Control (BOLD) dataset of echo i,
$S_{\mathrm{dd}}(\mathrm{TE}_i)$:	Dynamically divided (i.e., BOLD-corrected by computing $S_{\mathrm{bn}}(\mathrm{TE}_i)/S_{\mathrm{ctr}}(\mathrm{TE}_i)$) dataset of echo i,
δs:	Relative (i.e., percent) change in S,
ΔS:	Absolute change in S,
$\delta s_{\mathrm{BOLD,ctr}}(\mathrm{sum})$:	% BOLD signal change evaluated from $S_{ctr}(\mathrm{sum})$,
$\delta s_{\mathrm{BOLD,ctr}}(\mathrm{TE}_i)$:	% BOLD signal change evaluated from $S_{\mathrm{ctr}}(\mathrm{TE}_i)$,
$\delta s_{\mathrm{ctr}}(\mathrm{TE}\rightarrow 0)$:	% signal change (BOLD-like fluctuations) evaluated from $S_{\mathrm{ctr}}(\mathrm{TE}\rightarrow 0)$,
δs_{VASO}:	% VASO signal change (corrected or uncorrected for BOLD contamination)
$\delta s_{\mathrm{VASO,bn}}(\mathrm{TE}\rightarrow 0)$:	% VASO signal change, corrected for BOLD contamination by ME extrapolation i.e. evaluated from $S_{\mathrm{bn}}(\mathrm{TE}\rightarrow 0)$,
$\delta s_{\mathrm{VASO,bn}}(\mathrm{TE}_i)$:	% VASO signal change, uncorrected for BOLD contamination i.e. evaluated from $S_{\mathrm{bn}}(\mathrm{TE}_i)$,
$\delta s_{\mathrm{VASO,dd}}(\mathrm{TE}_i)$:	% VASO signal change, corrected for BOLD contamination by dynamic division i.e. evaluated from $S_{\mathrm{dd}}(\mathrm{TE}_i)$,
$T_{1,\mathrm{EV}}$:	EV (gray matter) longitudinal relaxation time, T_1 in ms,
$T_{2,\mathrm{EV}}^{*,\mathrm{rest}}$:	EV transverse relaxation time, T_2^* in ms as evaluated from resting state values of $R_{2,\mathrm{bn}}^*$ in the nulled slice,
$T_{2,\mathrm{EV+IV}}^{*,\mathrm{rest}}$:	Transverse relaxation time, T_2^* in ms as evaluated from resting state values of $R_{2,\mathrm{ctr}}^*$, hence based on signals from both EV and IV compartments,
$T_{2,\mathrm{IV}}^{*,\mathrm{rest}}$:	IV (blood) transverse relaxation time, T_2^* in ms,
TE:	Echo Time,
$\mathrm{TE}_{\mathrm{mid}}$:	TE corresponding to the middle echo of ME data,
TI:	Inversion Time,
V_{EV}:	Volume of EV compartment (gray matter) at rest,
$V_{\mathrm{EV+IV}}$:	Parenchymal (EV+IV) Volume at rest,
VASO:	Vascular Space Occupancy.

Supplementary Tables

Supplementary Table S1. Correlation of realignment parameters (translational and rotational motion) between VASO (S_{bn}) and control (S_{ctr}) scans for each readout.

Participants	Translational Motion		Rotational Motion		Mean correlation (r)	
	EPI	DEPICTING	EPI	DEPICTING	ME-EPI	DEPICTING
P1	0.93	0.94	0.96	0.87	0.95	0.91
P3	0.76	0.66	0.92	0.85	0.84	0.76
P4	0.93	0.77	0.61	0.96	0.77	0.87
P5	0.92	0.92	0.93	0.97	0.93	0.95
P9	0.91	0.79	0.80	0.81	0.86	0.80
P10	0.94	0.96	0.99	0.95	0.97	0.96
P11	0.87	0.66	0.87	0.69	0.87	0.68
P12	0.67	0.77	0.94	0.95	0.81	0.86
P13	0.77	0.98	0.99	0.93	0.88	0.96
P14	0.77	0.77	0.96	0.93	0.87	0.85
P16	0.81	0.86	0.69	0.86	0.75	0.86
**Average**	0.84	0.83	0.88	0.89	**0.86**	**0.86**

Supplementary Table S2. Number of significantly activated voxels (VASO and BOLD acquisitions) in all participants. The common ROI (last column), as used in further evaluations, was obtained by selecting all voxels showing VASO and BOLD activation on the, respectively, $S_{\mathrm{bn}}(\mathrm{TE}\rightarrow 0)$ and the $R^*_{2,\mathrm{ctr}}$ images obtained with both readouts. Sessions where ME-EPI scans preceded those of ME-DEPICTING have been marked with the letter 'e' following the participant ID, while those with ME-DEPICTING scans preceding ME-EPI are denoted with the letter 'd'.

Number of significant voxels at $p<10^{-6}$																
Par-tici-pants	VASO										BOLD		VASO ∩ BOLD			
	BOLD-uncorrected		Dynamic Division corrected						ME Extrapolated		Control Image		Commonly activated			
	$S_{\mathrm{bn}}(\mathrm{TE}_1)$		$S_{\mathrm{dd}}(\mathrm{TE}_1)$		$S_{\mathrm{dd}}(\mathrm{TE}_2)$		$S_{\mathrm{dd}}(\mathrm{TE}_3)$		$S_{\mathrm{bn}}(\mathrm{TE}\rightarrow 0)$		$R^*_{2,\mathrm{ctr}}$		$S_{\mathrm{bn}}(\mathrm{TE}\rightarrow 0) \cap R^*_{2,\mathrm{ctr}}$		ROI
	DPG	EPI	DPG	EPI	DPG	EPI	DPG	EPI	DPG	EPI	DPG	EPI	DPG	EPI	
P1e	710	258	1010	970	1205	1286	1191	1190	843	492	2219	2083	832	491	409
P3d	184	52	426	655	779	906	854	852	281	230	1865	1313	276	214	150
P4e	176	83	324	601	519	1011	555	868	274	241	1317	955	260	211	101
P5d	705	297	980	913	1290	1399	1350	1359	821	589	2065	1756	808	561	477
P9d	183	184	348	546	377	721	392	629	267	400	1594	1369	264	400	211
P10e	136	91	312	648	558	1076	633	982	241	233	1300	1317	241	232	156
P11d	414	201	720	635	856	922	946	940	473	302	1535	548	464	223	187
P12e	42	33	261	460	442	703	629	796	49	87	1216	1759	47	85	22
P13d	114	75	300	223	507	479	633	482	221	283	1481	1833	221	281	139
P14e	54	124	202	497	363	840	430	731	76	130	583	1146	70	127	41
P16e	255	335	412	875	564	1083	572	1067	324	455	1323	1541	315	450	257
**Mean ± SD**	**270 ± 238**	**158 ± 104**	**481 ± 287**	**638 ± 218**	**678 ± 320**	**948 ± 265**	**744 ± 308**	**900 ± 249**	**352± 263**	**313± 155**	**1500 ± 448**	**1420 ± 439**	**345 ± 260**	**298 ± 155**	**195 ± 136**

Supplementary Table S3. VASO CNR values over the common ROIs in each participant.

VASO CNR											
Participants	ROI	ME-EPI					ME-DEPICTING				
		$S_{\mathrm{bn}}(\mathrm{TE} \rightarrow 0)$	$S_{\mathrm{bn}}(\mathrm{TE}_1)$	$S_{\mathrm{dd}}(\mathrm{TE}_1)$	$S_{\mathrm{dd}}(\mathrm{TE}_2)$	$S_{\mathrm{dd}}(\mathrm{TE}_3)$	$S_{\mathrm{bn}}(\mathrm{TE} \rightarrow 0)$	$S_{\mathrm{bn}}(\mathrm{TE}_1)$	$S_{\mathrm{dd}}(\mathrm{TE}_1)$	$S_{\mathrm{dd}}(\mathrm{TE}_2)$	$S_{\mathrm{dd}}(\mathrm{TE}_3)$
P1	409	2.05	1.45	3.06	3.45	3.02	2.12	1.94	2.52	2.59	2.37
P3	126	1.36	0.71	1.71	2.04	1.83	1.36	1.09	1.38	1.42	1.34
P4	101	1.39	0.86	1.96	2.63	2.35	1.51	1.23	1.55	1.79	1.68
P5	477	1.60	1.13	2.14	2.72	2.66	1.79	1.61	2.09	2.38	2.50
P9	211	1.78	1.31	2.09	2.36	2.29	1.65	1.44	1.81	1.66	1.55
P10	156	1.68	0.9	2.79	3.18	2.98	1.63	1.23	1.84	2.38	2.47
P11	187	1.54	1.29	2.32	2.72	2.63	1.69	1.58	2.16	2.27	2.4
P12	22	1.58	1.29	2.63	2.98	2.92	1.29	1.22	1.82	1.94	2.05
P13	139	1.54	0.86	1.55	1.91	1.87	1.39	1.09	1.59	1.89	1.97
P14	41	1.34	1.26	1.93	2.48	2.28	1.3	1.2	1.62	1.72	1.73
P16	257	1.55	1.35	2.23	2.5	2.39	1.46	1.32	1.61	1.68	1.74
**Average ± SD**	**195± 136**	**1.60± 0.19**	**1.14 ±0.23**	**2.29± 0.43**	**2.69± 0.41**	**2.52± 0.36**	**1.59± 0.24**	**1.39± 0.25**	**1.87± 0.30**	**2.04± 0.33**	**2.05± 0.35**

Supplementary Table S4. BOLD CNR values over the common ROIs in each participant.

BOLD CNR											
Participants	ROI	ME-EPI					ME-DEPICTING				
		$S_{\mathrm{ctr}}(\mathrm{TE}_1)$	$S_{\mathrm{ctr}}(\mathrm{TE}_2)$	$S_{\mathrm{ctr}}(\mathrm{TE}_3)$	$S_{\mathrm{ctr}}(\mathrm{sum})$	$R^*_{2,\mathrm{ctr}}$	$S_{\mathrm{ctr}}(\mathrm{TE}_1)$	$S_{\mathrm{ctr}}(\mathrm{TE}_2)$	$S_{\mathrm{ctr}}(\mathrm{TE}_3)$	$S_{\mathrm{ctr}}(\mathrm{sum})$	$R^*_{2,\mathrm{ctr}}$
P1	409	1.37	1.75	1.76	5.52	5.12	2.52	2.59	2.37	4.17	3.68
P3	126	2.00	2.31	2.33	5.59	4.65	1.38	1.42	1.34	5.44	4.61
P4	101	1.68	2.27	2.27	4.19	3.91	1.55	1.79	1.68	3.64	3.58
P5	477	1.55	2.00	2.05	3.73	3.46	2.09	2.38	2.50	3.42	3.12
P9	211	1.32	1.77	1.76	3.03	2.83	1.81	1.66	1.55	3.17	3.21
P10	156	2.12	2.58	2.58	4.49	4.07	1.84	2.38	2.47	4.80	4.24
P11	187	2.09	2.54	2.59	2.48	2.44	2.16	2.27	2.4	3.04	2.85
P12	22	1.89	1.9	1.97	2.95	2.86	1.82	1.94	2.05	2.12	2.01
P13	139	1.75	2.2	2.28	5.7	5.5	1.59	1.89	1.97	3.87	3.82
P14	41	1.53	1.98	2.07	2.54	2.4	1.62	1.72	1.73	1.87	1.66
P16	257	1.96	2.3	2.33	3.16	3.11	1.61	1.68	1.74	2.58	2.57
**Average ± SD**	**195± 136**	**1.75± 0.28**	**2.15± 0.29**	**2.18± 0.29**	**3.9± 1.2**	**3.7± 1.1**	**1.08± 0.18**	**1.88± 0.27**	**2.02± 0.29**	**3.5± 1.1**	**3.2± 0.9**

Supplementary Table S5. Subject-wise corrected percent VASO signal change δs_{VASO}, from data extrapolated to zero TE, $S_{\text{bn}}(\text{TE} \to 0)$ and dynamically division data of all 3 echoes, $S_{\text{dd}}(\text{TE}_i)$ for both readouts.

δs_{VASO} % signal changes in commonly activated regions of both readouts									
Participants	ROI	ME-EPI				ME-DEPICTING			
		$S_{\text{bn}}(\text{TE} \to 0)$	$S_{\text{dd}}(\text{TE}_1)$	$S_{\text{dd}}(\text{TE}_2)$	$S_{\text{dd}}(\text{TE}_3)$	$S_{\text{bn}}(\text{TE} \to 0)$	$S_{\text{dd}}(\text{TE}_1)$	$S_{\text{dd}}(\text{TE}_2)$	$S_{\text{dd}}(\text{TE}_3)$
P1	409	-0.91	-1.19	-1.99	-2.37	-0.81	-0.99	-1.51	-2.00
P3	126	-0.74	-0.93	-1.49	-1.72	-0.54	-0.61	-0.86	-1.06
P4	101	-0.81	-1.02	-1.64	-1.94	-0.52	-0.58	-0.91	-1.22
P5	477	-0.87	-1.07	-1.75	-2.11	-0.67	-0.85	-1.25	-1.66
P9	211	-0.92	-1.01	-1.49	-1.65	-0.71	-0.84	-1.08	-1.19
P10	156	-0.61	-0.9	-1.56	-1.93	-0.33	-0.56	-0.96	-1.33
P11	187	-0.81	-1.06	-1.8	-2.28	-0.65	-0.85	-1.22	-1.71
P12	22	-0.62	-0.94	-1.39	-1.87	-0.39	-0.58	-0.86	-1.17
P13	139	-0.64	-0.73	-1.43	-1.79	-0.4	-0.49	-0.86	-1.21
P14	41	-0.74	-0.99	-1.47	-1.71	-0.44	-0.6	-0.84	-1.09
P16	257	-0.83	-1.14	-1.76	-2.14	-0.57	-0.71	-1.02	-1.37
**Average ± SD**	**195±136**	**-0.78±0.11**	**-1.02±0.13**	**-1.64±0.19**	**-1.98±0.23**	**-0.56±0.15**	**-0.71±0.16**	**-1.06±0.21**	**-1.40±0.28**

Supplementary Table S6. Subject-wise percent BOLD signal changes $\delta s_{\text{BOLD,ctr}}(\text{TE}_i)$ obtained from the control acquisitions, $S_{\text{ctr}}(\text{TE}_i)$ in the common ROI for both readouts.

$\delta s_{\text{BOLD,ctr}}$ % signal changes in commonly activated regions of both readouts							
Participants	ROI	ME-EPI			ME-DEPICTING		
		$S_{\text{ctr}}(\text{TE}_1)$	$S_{\text{ctr}}(\text{TE}_2)$	$S_{\text{ctr}}(\text{TE}_3)$	$S_{\text{ctr}}(\text{TE}_1)$	$S_{\text{ctr}}(\text{TE}_2)$	$S_{\text{ctr}}(\text{TE}_3)$
P1	409	0.43	1.49	2.21	0.26	1.06	2.05
P3	126	0.74	2.12	3.06	0.26	1.16	1.96
P4	101	0.56	1.86	2.76	0.17	0.99	1.87
P5	477	0.53	1.72	2.67	0.26	1.08	2.02
P9	211	0.46	1.47	2.16	0.20	0.89	1.50
P10	156	0.66	2.11	3.16	0.22	1.18	2.11
P11	187	0.53	1.73	2.68	0.28	0.95	1.81
P12	22	0.51	1.33	2.17	0.22	0.7	1.25
P13	139	0.48	1.68	2.62	0.21	1	1.95
P14	41	0.48	1.36	2.07	0.19	0.71	1.32
P16	257	0.59	1.7	2.52	0.22	0.82	1.51
**Average ± SD**	**195±136**	**0.54±0.09**	**1.69±0.27**	**2.55±0.37**	**0.23±0.03**	**0.96±0.16**	**1.76±0.31**

Supplementary Figures

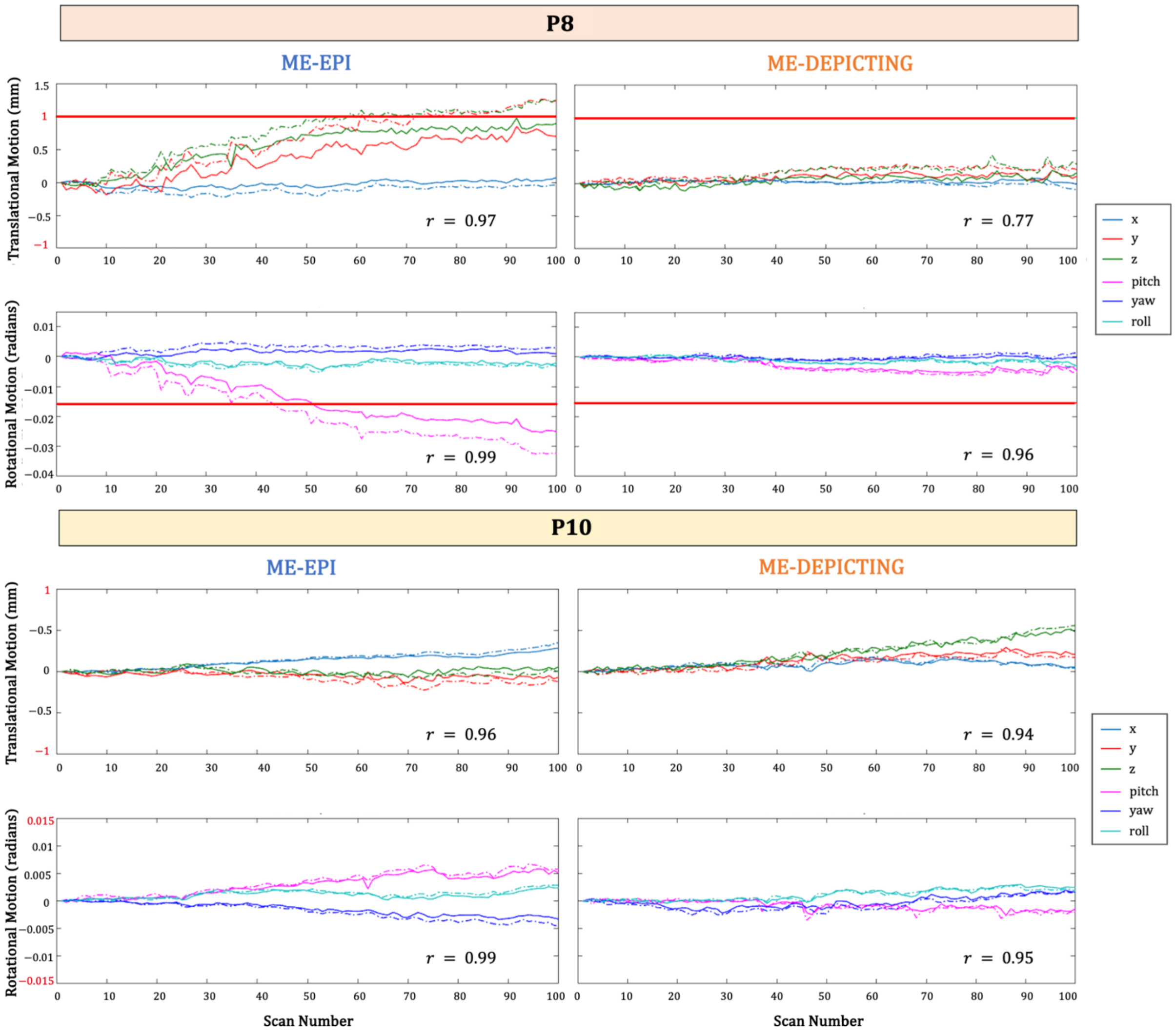

Supplementary Figure S1. Example realignment (motion) parameters obtained from SPM12 pre-processing in an excluded (P8) and included (P10) participant. Subject motion was calculated based on the first volume of the TE_1 data. Translational motion is given in terms of displacement in mm while rotational motion is given in terms of radians over the x-, y- and z-axes (pitch: nodding, yaw: shaking and roll: titling). Solid lines represent displacement of the blood-nulled (S_{bn}) images while the dotted lines denote displacement of the control (S_{ctr}) images over time (scans). Their mutual correlation coefficient, r, is also provided. The bold red lines in the plots for P8 represent the pre-set motion thresholds. Note that ME-DEPICTING scans from this particular session were also excluded to allow for a sequence comparison. The plots for P10 are well within the thresholds.

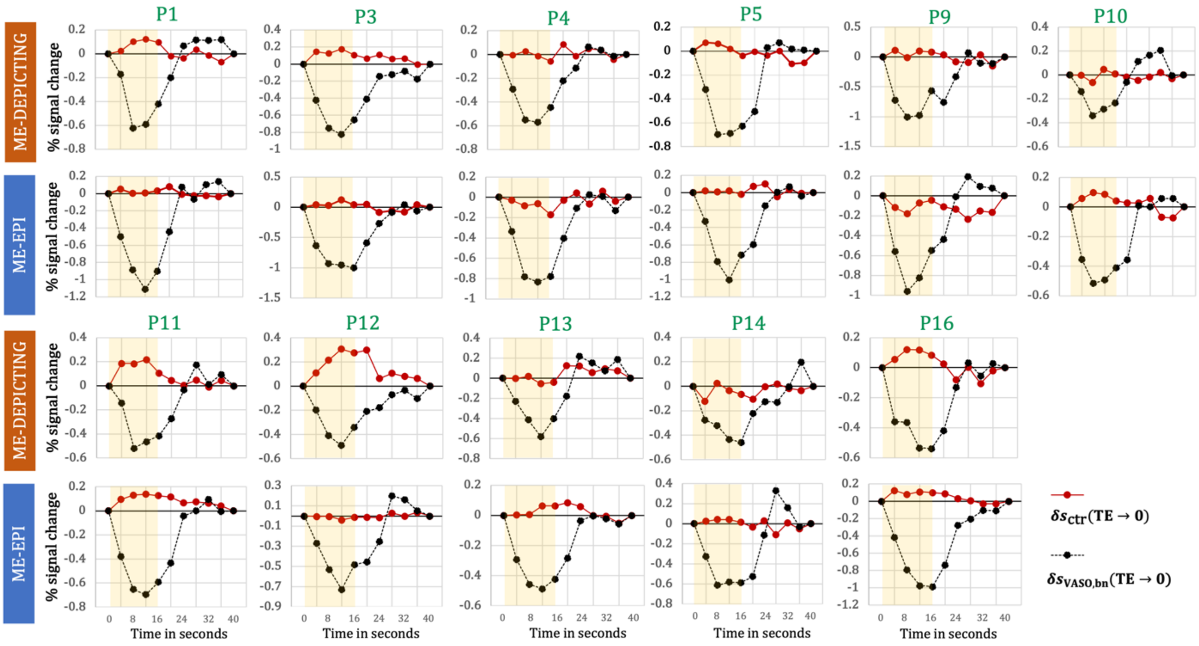

Supplementary Figure S2. Fluctuations in the ME extrapolated control image. $\delta s_{ctr}(TE\rightarrow0)$ signals (averaged over the functional cycle) as obtained from the $S_{ctr}(TE \rightarrow 0)$ images of ME-DEPICTING and ME-EPI have been plotted individually for all 11 participants (in red). Time courses of $\delta s_{VASO,bn}(TE\rightarrow0)$ for both readouts are also provided for reference (dotted black). The task duration is shaded in yellow. All timecourses were extracted from ROIs defined by significant VASO activation from $S_{bn}(TE \rightarrow 0)$ images of the two readouts (ME-Extrapolated column in Table S2) for each participant.

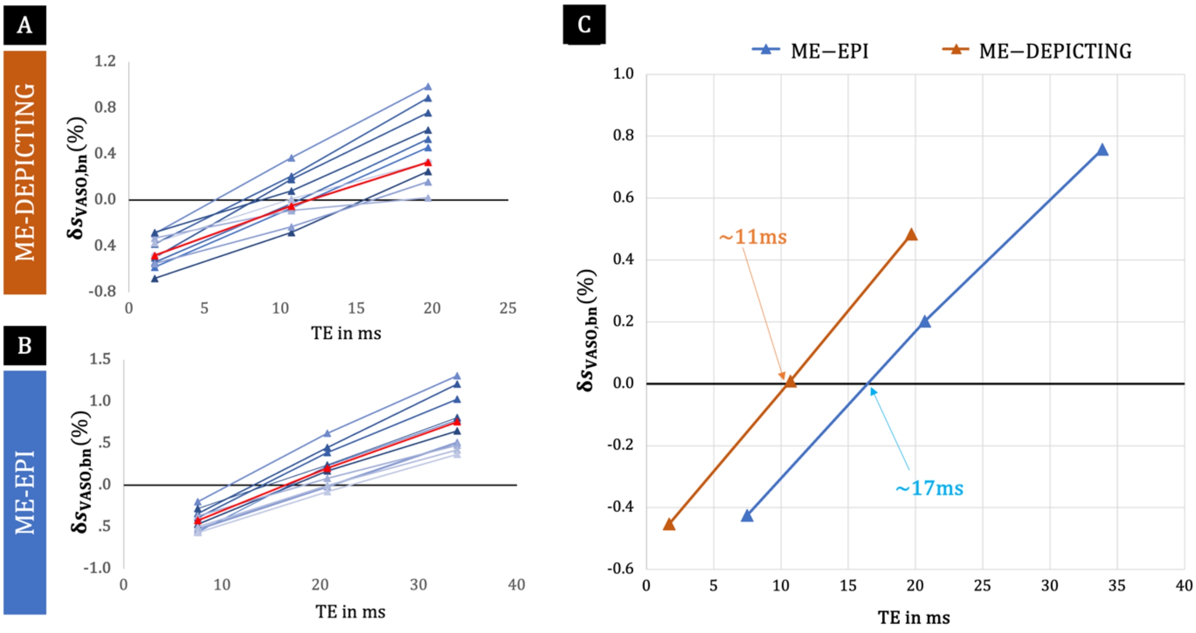

Supplementary Figure S3. Zero crossing of BOLD uncorrected VASO signal changes. Echo wise plot of the mean $\delta s_{VASO,bn}(TE_i)$ over respective TE_i for ME-DEPICTING (A) and ME-EPI (B) for all participants. Subject-averaged values are highlighted in red. (C) Subject averaged plots in (A) & (B) replotted for the two readouts. The echo times corresponding to the zero-crossing of $\delta s_{VASO,bn}$ for ME-DEPICTING and ME-EPI are indicated, respectively, with orange and with blue arrows.
